# Prenatal exposure to a wide range of environmental chemicals and child behaviour between 3 and 7 years of age – An exposome-based approach in 5 European cohorts

**DOI:** 10.1016/j.scitotenv.2020.144115

**Published:** 2021-04-01

**Authors:** Paulina Jedynak, Léa Maitre, Mónica Guxens, Kristine B. Gützkow, Jordi Julvez, Mónica López-Vicente, Jordi Sunyer, Maribel Casas, Leda Chatzi, Regina Gražulevičienė, Mariza Kampouri, Rosie McEachan, Mark Mon-Williams, Ibon Tamayo, Cathrine Thomsen, José Urquiza, Marina Vafeiadi, John Wright, Xavier Basagaña, Martine Vrijheid, Claire Philippat

**Affiliations:** a University Grenoble Alpes, Inserm, CNRS, Team of Environmental Epidemiology applied to Reproduction and Respiratory Health, Institute for Advanced Biosciences (IAB), Grenoble, France; b ISGlobal, Barcelona, Spain; c Universitat Pompeu Fabra (UPF), Barcelona, Spain; d CIBER Epidemiología y Salud Pública (CIBERESP), Madrid, Spain; e Department of Child and Adolescent Psychiatry/Psychology, Erasmus Medical Centre–Sophia Children's Hospital, Rotterdam, the Netherlands; f Norwegian Institute of Public Health, Oslo, Norway; g Institut d'Investigació Sanitària Pere Virgili (IISPV), Hospital Universitari Sant Joan de Reus, Reus, Spain; h Department of Preventive Medicine, Keck School of Medicine, University of Southern California, Los Angeles, CA, USA; i Department of Social Medicine, University of Crete, Heraklion, Greece; j Department of Genetics and Cell Biology, Faculty of Health, Medicine and Life Sciences, Maastricht University, Maastricht, Netherlands; k Department of Environmental Sciences, Vytautas Magnus University, Kaunas, Lithuania; l Bradford Institute for Health Research, Bradford Teaching Hospitals NHS Foundation Trust, Bradford, UK

**Keywords:** Internal exposome, Prenatal exposure, Child behaviour, Strengths and Difficulties Questionnaire, Birth cohort

## Abstract

**Background:**

Studies looking at associations between environmental chemicals and child behaviour usually consider only one exposure or family of exposures.

**Objective:**

This study explores associations between prenatal exposure to a wide range of environmental chemicals and child behaviour.

**Methods:**

We studied 708 mother-child pairs from five European cohorts recruited in 2003–2009. We assessed 47 exposure biomarkers from eight chemical exposure families in maternal blood or urine collected during pregnancy. We used the Strengths and Difficulties Questionnaire (SDQ) to evaluate child behaviour between three and seven years of age. We assessed associations of SDQ scores with exposures using an adjusted least absolute shrinkage and selection operator (LASSO) considering all exposures simultaneously and an adjusted exposome-wide association study (ExWAS) considering each exposure independently.

**Results:**

LASSO selected only copper (Cu) as associated with externalizing behaviour. In the ExWAS, bisphenol A [BPA, incidence rate ratio (IRR): 1.06, 95% confidence interval (95%CI): 1.01;1.12] and mono-n-butyl phthalate (MnBP, IRR: 1.06, 95%CI: 1.00;1.13) were associated with greater risk of externalizing behaviour problems. Cu (IRR: 0.90, 95%CI: 0.82;0.98), perfluoroundecanoate (PFUnDA, IRR: 0.92, 95%CI: 0.84;0.99) and organochlorine compounds (OCs) were associated with lower risk of externalizing behaviour problems, however the associations with OCs were mainly seen among women with insufficient weight gain during pregnancy. Internalizing score worsen in association with exposure to diethyl thiophosphate (DETP, IRR: 1.11, 95%CI: 1.00;1.24) but the effect was driven by the smallest cohort. Internalizing score improved with increased concentration of perfluorooctane sulfonate (PFOS, IRR: 0.92, 95%CI: 0.85;1.00), however the association was driven by the two smallest cohorts with the lowest PFOS concentrations.

**Discussion:**

This study added evidence on deleterious effects of prenatal exposure to BPA and MnBP on child behaviour. Other associations should be interpreted cautiously since they were not consistent with previous studies or they have not been studied extensively.

## Highlights

• We studied simultaneous effect of prenatal exposure to several compounds on child behaviour.
• 47 exposure biomarkers from 8 chemical exposure families were studied in 5 European cohorts.
• Bisphenol A and mono-n-butyl phthalate were associated with increased behavioural problems in children.
• Copper was associated with decreased behavioural problems.

## Graphical abstract

**Figure:**
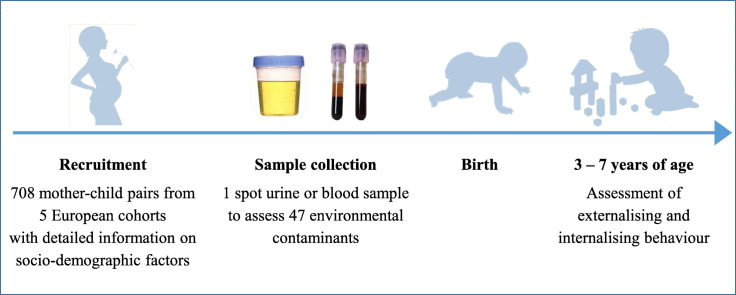

## 1 Introduction

Child neurodevelopmental disorders are associated with long-term functional impairments which cause substantial social and financial costs for the affected individuals, their families and society as a whole. The annual cost (including medical and non-medical costs) of child neurodevelopment disorders in Europe has been estimated at €21 billion (Gustavsson et al., 2011). This makes the identification of modifiable risk factors for these disorders a priority target for public health. The root causes of most childhood neurodevelopmental disorders are multifactorial and only partly understood. In addition to genetic factors, exposure to environmental contaminants⁠ during periods of high sensitivity of the brain, such as pregnancy and early childhood, is suspected to play a role in the origin of neurodevelopmental disorders (Bellinger, 2009; Grandjean and Landrigan, 2014). In a review focusing on human studies, Grandjean and Landrigan identified 12 environmental chemicals or families of chemicals as neurodevelopmental toxicants (Grandjean and Landrigan, 2006, Grandjean and Landrigan, 2014), including several metals and inorganic compounds (lead, methylmercury, inorganic arsenic, manganese, fluoride), polychlorinated biphenyls (PCBs), some solvents (toluene, ethanol), certain pesticides [organophosphate (OP) pesticides] and polybrominated diphenyl ethers (PBDEs). The authors listed over 200 additional chemicals, including some phthalates, bisphenols, and cotinine, that are potentially neurotoxic in humans based on data from the US National Library of Medicine, the US Agency for Toxic Substances and Disease Registry, and the US Environmental Protection Agency.

With few exceptions (e.g., Braun et al., 2014; Kim et al., 2018; Maitre et al. submitted to journal; Tanner et al., 2020), epidemiological studies analysing the effects of environmental contaminants on child neurodevelopment have considered only one exposure or family of exposures, while in real life individuals are exposed to a wide range of environmental compounds that could simultaneously affect development and health (Haug et al., 2018). Studies considering several exposures simultaneously are needed to improve the understanding of the potential effects of environmental risk factors on neurodevelopmental disorders and ameliorate their prevention (Siroux et al., 2016). The aim of this study was to assess the associations between prenatal exposure to a wide range of environmental chemicals (*n* = 47) and child behaviour.

## 2 Methods

### 2.1 Study design and population

This study is a part of the HELIX project which includes six European mother-child cohorts: Born in Bradford (BiB, UK), Étude des Déterminants Pré et Postnatals du Développement et de la Santé de l'Enfant (EDEN, France), Infancia y Medio Ambiente (INMA, Spain), Kaunas Cohort (KANC, Lithuania), Norwegian Mother, Father and Child Cohort Study (MoBa, Norway) and Mother-Child Cohort in Crete (RHEA, Greece). The study design is described in detail elsewhere (Maitre et al., 2018; Vrijheid et al., 2014). Out of the 1301 children originally included in the HELIX sub-cohort (Maitre et al., 2018; Vrijheid et al., 2014), we relied on a sub-sample of 708 mother-child pairs for which child behaviour was assessed using the Strengths and Difficulties Questionnaire (SDQ) at three to seven years of age (Appendix Fig. 1). Children from the MoBa cohort were not included because the SDQ was not implemented in this group.

### 2.2 Assessment of prenatal exposure to environmental chemicals

We assessed 54 biomarkers of exposure to a broad spectrum of environmental chemicals (Appendix Table 1, Appendix Table 2). Briefly, in blood we assessed biomarkers of exposure to eight organochlorine compounds (OCs), two PBDEs, five per- and polyfluoroalkyl substances (PFASs) and 15 metals and non-metals (essential and toxic elements). In urine, we assessed biomarkers of exposure to 10 phthalate metabolites, seven phenols, six OP pesticide metabolites, and cotinine. Out of those, we excluded five essential elements not considered to be neurotoxic as well as thallium and diethyl dithiophosphate due to their low frequency of detection (1.5% and 2.1%, respectively). This left 47 biomarkers for further analyses. Methods of biomarker assessment and descriptive statistics and correlation patterns between the biomarkers are described elsewhere (Haug et al., 2018; Tamayo-Uria et al., 2019).

### 2.3 Behavioural outcomes

We evaluated child behaviour using the SDQ (Goodman, 1997), which was completed by the mothers between three and seven years of child's age. SDQ scores were collected as part of the individual cohort initiatives and harmonized and pooled a posteriori. In this analysis we relied on the combined externalizing and internalizing scores only, since they have been shown to be more consistent across informants (e.g., parents, teachers) and more discriminant with respect to clinical disorders in low-risk community samples, like the one examined in our study, compared to the five sub-scales (Goodman et al., 2010) (Appendix Table 3). Moreover, given our limited sample size and the large number of studied exposure biomarkers, combining the SDQ sub-scales limited the number of performed tests.

### 2.4 Statistical analysis

We singly imputed biomarker concentrations below the limit of detection using a quantile regression approach for the imputation of left-censored missing data (Nadarajah and Kotz, 2006). We divided urinary biomarker concentrations by creatinine concentration. Haemal lipophilic biomarker concentrations were standardized and expressed in ng/g of total lipids in serum or plasma. Concentrations were then ln-transformed (cotinine) or log_2_-transformed (all other biomarkers) to approach normality and standardized for the interquartile range (IQR) by dividing biomarker concentration observed for each individual for a given exposure by the IQR calculated for this exposure.

We selected the following adjustment factors based on a priori knowledge: cohort, season of conception, child's sex and age at the SDQ assessment, parity, maternal age and education level, maternal working and active smoking status during pregnancy and maternal pre-pregnancy body mass index (see Appendix Table 4 for details). Missing data for exposure biomarker concentrations (see Appendix Table 5 for details) and adjustment factors were multiply imputed (100 imputed datasets) via a chained equations algorithm (White et al., 2011). To explore the associations between 47 biomarkers and externalizing and internalizing behaviour scores we applied two statistical approaches. First, we used a least absolute shrinkage and selection operator (LASSO) algorithm with log link function. LASSO considers all exposures simultaneously (Tibshirani, 1996) and performs variable selection through estimates' shrinkage (i.e., the lowest regression coefficients corresponding to the least informative predictors are assigned a zero value). We determined the range of penalty parameter λ by maximizing the prediction log-likelihood using 10-fold cross-validation. To prevent overfitting, we defined the optimal λ as the one providing the sparsest model (as measured by the number of nonzero regression coefficients) among those yielding a log-likelihood within one standard error of the maximum log-likelihood (Krstajic et al., 2014). To stabilise estimates, LASSO was fit on each of the 100 imputed datasets and an exposure was retained only if it was selected in at least 50% of runs (Wood et al., 2008). Second, to compare with previous single-pollutant studies, we also performed an exposome-wide association study (ExWAS): we fit a negative binomial regression model on each of the 100 imputed datasets for each exposure biomarker and SDQ score, then aggregated the results using Rubin's rule for multiply imputed data (Patel et al., 2010). To control for multiple comparisons, we applied a family-wise error rate (FWER) correction to the p value threshold. The correction uses a Bonferroni procedure extended to handle correlated tests: the actual number of exposures being tested (M) is replaced by a smaller value called the effective number of independent exposures (M_e_). M_e_ is estimated by ∑_*i*=1_^*M*^[*I*(*λ*_*i*_ > 1)(*λ*_*i*_ − 1)], where I(x) is an indicator function and λ_i_ are the eigenvalues of the matrix of correlations between M exposures. The p value threshold to control FWER to α, using M_e_ in a Bonferroni procedure, is then α / M_e_ (adapted from Li et al., 2012).

To test the robustness of the associations between SDQ scores and exposure biomarkers identified by the LASSO (selected in at least 50% of runs) and ExWAS (those with uncorrected p values <0.05) we performed further sensitivity analyses. We evaluated the linearity of the associations using generalized additive model (GAM) with restricted cubic splines function. Then we ran a regression simultaneously adjusted for all biomarkers associated with the SDQ scores in the main ExWAS (p values <0.2). We additionally adjusted our main model for breastfeeding and fish and seafood consumption during pregnancy (since fish and seafood may accumulate persistent organic contaminants and heavy metals). We explored sex-specific effects by adding an interaction term between each biomarker of exposure and child sex and performed an ExWAS restricted to the participants with no missing biomarker concentrations. For the biomarkers associated with the SDQ externalizing score we ran an ExWAS after exclusion of the BiB cohort, as we had noted that children from this population had markedly lower externalizing score (median = 0.5) compared to the other cohorts (medians ≥5, Table 1). Apart from the mentioned analyses, for all measured exposure biomarkers we evaluated the between-cohort heterogeneity of the adjusted association using the I^2^ statistic (Higgins and Thompson, 2002). We relied on the following threshold for the I^2^ interpretation: I^2^ < 0.3: low heterogeneity, 0.3 ≤ I^2^ < 0.6: moderate heterogeneity, I^2^ ≥ 0.6: substantial to high heterogeneity (Deeks et al., 2019). Finally, because excessive maternal weight gain during pregnancy could lead to decreased blood concentrations of lipophilic compounds due to their storage in the adipose tissue (Kim et al., 2011; Lee et al., 2014; Verner et al., 2013) and to behavioural problems in the offspring (Pugh et al., 2016), we ran an additional analysis stratified on gestational weight gain for all the biomarkers from the OCs family.

**Table 1:** Population characteristics for the mother-child pairs included in the study: overall and by cohort.

	Overall distribution	Cohort-specific distribution					p value of equality between cohorts^a^
		BiB	EDEN	INMA	KANC	RHEA	
		46 (6.5%)	193 (27.3%)	218 (30.8%)	83 (11.7%)	168 (23.7%)	
Season of conception	··	··	··	··	··	··	<0.001
January-March	208 (29.4%)	21 (45.7%)	65 (33.7%)	47 (21.6%)	26 (31.3%)	49 (29.2%)	
April-June	159 (22.5%)	5 (10.9%)	41 (21.2%)	49 (22.5%)	10 (12.0%)	54 (32.1%)	
July-September	174 (24.6%)	11 (23.9%)	34 (17.6%)	61 (28.0%)	29 (34.9%)	39 (23.2%)	··
October-December	164 (23.2%)	9 (19.6%)	53 (27.5%)	61 (28.0%)	16 (19.3%)	25 (14.9%)	··
Missing	3 (0.4%)	0 (0.0%)	0 (0.0%)	0 (0.0%)	2 (2.4%)	1 (0.6%)	··
Active smoking during pregnancy	··	··	··	··	··	··	<0.001
No	553 (78.1%)	35 (76.1%)	147 (76.2%)	162 (74.3%)	77 (92.8%)	132 (78.6%)	··
Yes	145 (20.5%)	6 (13.0%)	46 (23.8%)	54 (24.8%)	4 (4.8%)	35 (20.8%)	··
Missing	10 (1.4%)	5 (10.9%)	0 (0.0%)	2 (0.9%)	2 (2.4%)	1 (0.6%)	··
Parity	··	··	··	··	··	··	<0.001
Nulliparous	317 (44.8%)	20 (43.5%)	89 (46.1%)	117 (53.7%)	27 (32.5%)	64 (38.1%)	··
1 child	268 (37.9%)	15 (32.6%)	70 (36.3%)	90 (41.3%)	26 (31.3%)	67 (39.9%)	··
≥ 2 children	114 (16.1%)	10 (21.7%)	34 (17.6%)	10 (4.6%)	28 (33.7%)	32 (19.0%)	··
Missing	9 (1.3%)	1 (2.2%)	0 (0.0%)	1 (0.5%)	2 (2.4%)	5 (3.0%)	··
Maternal level of education	··	··	··	··	··	··	<0.001
Primary school	89 (12.6%)	18 (39.1%)	12 (6.2%)	52 (23.9%)	2 (2.4%)	5 (3.0%)	··
Secondary school	292 (41.2%)	8 (17.4%)	71 (36.8%)	91 (41.7%)	32 (38.6%)	90 (53.6%)	··
University degree or higher	317 (44.8%)	17 (37.0%)	108 (56.0%)	74 (33.9%)	47 (56.6%)	71 (42.3%)	··
Missing	10 (1.4%)	3 (6.5%)	2 (1.0%)	1 (0.5%)	2 (2.4%)	2 (1.2%)	··
Maternal work status	··	··	··	··	··	··	<0.001
Unemployed	128 (18.1%)	13 (28.3%)	31 (16.1%)	18 (8.3%)	13 (15.7%)	53 (31.5%)	··
Employed	560 (79.1%)	22 (47.8%)	162 (83.9%)	197 (90.4%)	68 (81.9%)	111 (66.1%)	··
Missing	20 (2.8%)	11 (23.9%)	0 (0.0%)	3 (1.4%)	2 (2.4%)	4 (2.4%)	··
Maternal pre-pregnancy BMI	··	··	··	··	··	··	<0.001
Underweight	28 (4.0%)	0 (0.0%)	16 (8.3%)	9 (4.1%)	1 (1.2%)	2 (1.2%)	··
Normal weight	426 (60.2%)	13 (28.3%)	120 (62.2%)	149 (68.3%)	29 (34.9%)	115 (68.5%)	··
Overweight	158 (22.3%)	17 (37.0%)	39 (20.2%)	41 (18.8%)	28 (33.7%)	33 (19.6%)	··
Obesity	87 (12.3%)	14 (30.4%)	16 (8.3%)	19 (8.7%)	23 (27.7%)	15 (8.9%)	··
Missing	9 (1.3%)	2 (4.3%)	2 (1.0%)	0 (0.0%)	2 (2.4%)	3 (1.8%)	··
Gestational weight gain based on maternal pre-pregnancy BMI^b^	··	··	··	··	··	··	<0.001
Insufficient	186 (26.3%)	7 (15.2%)	51 (26.4%)	76 (34.9%)	14 (16.9%)	38 (22.6%)	··
Adequate	199 (28.1%)	10 (21.7%)	63 (32.6%)	62 (28.4%)	14 (16.9%)	50 (29.8%)	··
Excessive	263 (37.1%)	10 (21.7%)	59 (30.6%)	74 (33.9%)	45 (54.2%)	75 (44.6%)	··
Missing	60 (8.5%)	19 (41.3%)	20 (10.4%)	6 (2.7%)	10 (12.0%)	5 (3.0%)	··
Child sex^c^	··	··	··	··	··	··	0.860
Female	313 (44.2%)	18 (39.1%)	83 (43.0%)	102 (46.8%)	35 (42.2%)	75 (44.6%)	··
Male	395 (55.8%)	28 (60.9%)	110 (57.0%)	116 (53.2%)	48 (57.8%)	93 (55.4%)	··
Missing	0 (0.0%)	0 (0.0%)	0 (0.0%)	0 (0.0%)	0 (0.0%)	0 (0.0%)	··
Child age at SDQ assessment (years)^c^	5.6 [4.2;6.4]	5.4 [5.1;5.5]	5.6 [5.5;5.7]	6.8 [6.5;6.9]	4.5 [4.1;4.9]	4.1 [4.1;4.2]	<0.001
Missing	0 (0.0%)	0 (0.0%)	0 (0.0%)	0 (0.0%)	0 (0.0%)	0 (0.0%)	··
Maternal age (years)	30.9 [27.7;34.1]	29.5 [22.2;34.0]	30.0 [27.6;34.0]	32.1 [29.5;34.7]	29.8 [26.8;32.7]	31.0 [27.3;34.0]	<0.001
Missing	4 (0.6%)	0 (0.0%)	0 (0.0%)	0 (0.0%)	2 (2.4%)	2 (1.2%)	··
SDQ externalizing score^c^	5.0 [2.8;7.0]	0.5 [0.0;3.8]	5.0 [2.0;7.0]	5.0 [3.0;8.0]	6.0 [4.0;8.5]	5.0 [3.0;7.0]	<0.001
Missing	0 (0.0%)	0 (0.0%)	0 (0.0%)	0 (0.0%)	0 (0.0%)	0 (0.0%)	··
SDQ internalizing score^c^	3.0 [1.0;5.0]	2.0 [0.0;4.0]	3.0 [1.0;5.0]	3.0 [1.0;4.8]	3.0 [2.0;5.0]	3.0 [1.0;4.0]	0.012
Missing	0 (0.0%)	0 (0.0%)	0 (0.0%)	0 (0.0%)	0 (0.0%)	0 (0.0%)	··

Distributions are reported as number and percentage for categorical variables and as median, 1^st^ and 3^rd^ quartiles for continuous variables. All values are before imputation.

a Kruskal-Wallis test was applied on continuous variables and χ² or exact Fisher test was applied on categorical variables.

b Gestational weight gain based on maternal pre-pregnancy BMI was categorized into 3 categories: insufficient, adequate and excessive according to the recommendations of the US Institute of Medicine (Institute of Medicine (US) and National Research Council (US) Committee to Reexamine IOM Pregnancy Weight Guidelines 2009). For BMI < 18.5 kg/m^2^ recommended total weight gain was 12.5-18.0 kg, for BMI = 18.5-24.9 kg/m^2^: 11.5-16.0 kg, for BMI = 25.0-29.9 kg/m^2^: 7.0-11.5 kg and for BMI ≥ 30.0 kg/m^2^ recommended weight gain was 5.0-9.0 kg.

c Child sex, child age at the SDQ assessment and SDQ scores were not imputed. Abbreviations: BiB = Born in Bradford. EDEN = Étude des Déterminants Pré et Postnatals du Développement et de la Santé de l’Enfant. INMA = Infancia y Medio Ambiente. KANC = Kaunas Cohort. RHEA = Mother-Child Cohort in Crete. BMI = body mass index. SDQ = Strengths and Difficulties Questionnaire.

All analyses were conducted using R v. 4.0.2 (R Core Team and R Foundation for Statistical Computing, 2020)⁠ and RStudio v. 1.3.1056 (RStudio Team, 2020) using packages: *mice* (van Buuren and Groothuis-Oudshoorn, 2011)⁠ for multiple imputation, *mpath* (Wang et al., 2015) to fit LASSO, *MASS* (Venables and Ripley, 2002) for the ExWAS analysis, *metaplus* (Beath, 2016) to estimate between-cohort heterogeneity and *gam* (Hastie, 2020) and *rms* (Harrell Jr, 2020) to evaluate linearity of associations between biomarkers of exposure and SDQ scores.

Data used in this study is confidential and can only be provided upon request and after approval of the HELIX consortium. The code is available in the public repository of the Team of Environmental Epidemiology applied to Reproduction and Respiratory Health (https://gricad-gitlab.univ-grenoble-alpes.fr/iab-env-epi).

## 3 Results

### 3.1 Characteristics of the study population and prenatal exposure to environmental contaminants

Characteristics of the study population and exposure biomarker distributions are detailed in Table 1 and Appendix Table 5, respectively. Median child age at the SDQ assessment was 5.6 years. Median SDQ externalizing and internalizing scores were 5 and 3 points, respectively. Heterogeneity was observed between cohorts for most covariates as well as for the SDQ scores, with parents from the BiB cohort reporting behaviour scores of their children to be better than of those from other cohorts (p value of the Kruskal-Wallis test <0.001, Table 1). High frequency of detection was observed for most of the 47 exposure biomarkers, with 39 detected in at least 89% of the tested samples (Appendix Table 5). Heterogeneity was observed between cohorts for most exposures (p values of the Kruskal-Wallis test <0.05, Appendix Table 5).

### 3.2 Association between prenatal chemical exposome and SDQ scores

#### 3.2.1 Externalizing score

Among the 47 exposures studied, the adjusted LASSO for the externalizing score selected only copper (Cu). Cu was also detected in the ExWAS analysis as associated with lower externalizing score, meaning decreased risk of behavioural problems [Incidence rate ratio (IRR): 0.90, 95% confidence interval (CI): 0.82;0.98 for an IQR change in the log_2_-transformed Cu concentration, Table 2]. In addition to Cu, the ExWAS identified five other associations. Bisphenol A (BPA, IRR: 1.06, 95%CI: 1.01;1.12) and mono-n-butyl phthalate (MnBP, IRR: 1.06, 95%CI: 1.00;1.13) were positively associated with the externalizing score, while perfluoroundecanoate (PFUnDA, IRR: 0.92, 95%CI: 0.84;0.99) and two OCs [dichlorodiphenyltrichloroethane (DDT, IRR: 0.92, 95%CI: 0.84;1.00) and PCB-138 (IRR: 0.88, 95%CI: 0.79;0.99)] were negatively associated with this score. While not significant (p values ranged between 0.065 for PCB-153 to 0.253 for PCB-180), all the other compounds from the OCs family tended to be negatively associated with the externalizing score (Appendix Table 6).

**Table 2:** Adjusted associations^a^ between the prenatal exposure to environmental contaminants and SDQ externalizing and internalizing scores (*n* = 708 unless stated otherwise).

Behavioural outcome	Exposure	Exposure family	ExWAS^a^			Model simultaneously adjusted for coexposures^b^		ExWAS for complete case analysis			ExWAS after exclusion of the BiB cohort^c^		
			IRR (95%CI)^d^	p value	FWER p value	IRR (95%CI)^d^	p value	IRR (95%CI)^d^	p value	n	IRR (95%CI)^d^	p value	n
SDQ externalizing score	BPA (μg/g of creatinine)	Phenol	1.06 (1.01; 1.12)	0.028	0.842	1.05 (0.99; 1.11)	0.105	1.07 (1.01; 1.13)	0.013	580	1.06 (1.01; 1.12)	0.026	662
	Cu (μg/L of whole blood)	Essential element	0.90 (0.82; 0.98)	0.021	0.631	0.90 (0.82; 0.99)	0.030	0.92 (0.85; 0.99)	0.031	314	0.91 (0.83; 1.00)	0.042	662
	DDT (ng/g of lipids)	Organochlorine compound	0.92 (0.84; 1.00)	0.045	1	0.94 (0.85; 1.03)	0.163	0.87 (0.79; 0.97)	0.008	356	0.94 (0.86; 1.03)	0.174	662
	MnBP (μg/g of creatinine)	Phthalate	1.06 (1.00; 1.13)	0.048	1	1.06 (1.00; 1.13)	0.058	1.07 (1.00; 1.14)	0.046	585	1.06 (1.00; 1.13)	0.041	662
	PCB-138 (ng/g of lipids)	Organochlorine compound	0.88 (0.79; 0.99)	0.035	1	0.95 (0.82; 1.11)	0.539	0.86 (0.75; 0.98)	0.030	567	0.88 (0.79; 0.99)	0.031	662
	PFUnDA (μg/L of serum, plasma or whole blood)	Perfluoroalkyl substance	0.92 (0.84; 0.99)	0.034	1	0.94 (0.87; 1.02)	0.164	0.95 (0.87; 1.03)	0.212	447	0.90 (0.82; 0.98)	0.013	662
SDQ internalizing score	DETP (μg/g of creatinine)	OP pesticide metabolite	1.11 (1.00; 1.24)	0.053	1	1.11 (0.99; 1.23)	0.067	1.09 (0.97; 1.23)	0.141	560			
	PFOS (μg/L of serum, plasma or whole blood)	Perfluoroalkyl substance	0.92 (0.85; 1.00)	0.037	1	0.94 (0.81; 1.08)s	0.381	0.93 (0.86; 1.01)	0.091	646			

a Only associations with uncorrected p values < 0.05 (except for DETP with the p value = 0.053) in the main ExWAS are displayed in this table.

b Results from a multiple linear regression model including all exposures with p value < 0.2 in the main ExWAS. Due to the high correlation with PCB-138 (Spearman's rho = 0.97, variation inflation factor = 18.7), PCB-153 was excluded from the SDQ externalizing coexposure. The models adjusted for coexposures included 13 biomarkers for the SDQ externalizing score (BPA, Cd, Co, Cu, DDE, DDT, HCB, MnBP, PBDE-47, PCB-118, PCB-138, PFUnDA and PRPA) and 8 for the SDQ internalizing score (BUPA, Co, DETP, Mn, PFHxS, PFNA, PFOS and PFUnDA). In addition to the mentioned biomarkers, regression models were adjusted for cohort, season of conception, child sex and age at SDQ assessment, parity, maternal: education level, work status, age, pre-pregnancy BMI, and prenatal active smoking status.

c The analysis was performed only for the SDQ externalizing score.

d IRRs are reported with 95%CIs and correspond to the change in the probability of the SDQ scores increasing by one unit for an IQR change in the log_2_ of the biomarker concentration in maternal blood or urine. Abbreviations: BiB = Born in Bradford. BMI = body mass index. CI = confidence interval of the IRR estimate. ExWAS = exposome-wide association study. FWER = family wise error rate adjustment. IQR = inter-quartile range. IRR = incident rate ratio. SDQ = Strengths and Difficulties Questionnaire. BPA = bisphenol A. BUPA = n-butyl paraben. Cd = cadmium. Co = cobalt. Cu = copper. DDE = dichlorodiphenyldichloroethylene. DDT = dichlorodiphenyltrichloroethane. DETP = diethyl thiophosphate. HCB = hexachlorobenzene. Mn = manganese. MnBP = mono-n-butyl phthalate. OP = organophosphate. PBDE-47 = polybrominated diphenyl ether-47. PCB = polychlorinated biphenyl. PFHxS = perfluorohexane sulfonate. PFNA = perfluorononanoate. PFOS = perfluorooctane sulfonate. PFUnDA = perfluoroundecanoate. PRPA = propyl paraben.

#### 3.2.2 Internalizing score

The adjusted LASSO did not retain any exposure biomarker as being associated with the internalizing score, while the ExWAS identified a positive association with diethyl thiophosphate (DETP) concentration close to the significance level (IRR: 1.11, 95%CI: 1.00;1.24) and a negative association with perfluorooctane sulfonate concentration (PFOS, IRR: 0.92, 95%CI: 0.85;1.00, Table 2 and Appendix Table 7).

#### 3.2.3 Sensitivity analyses

**Fig. 1:**
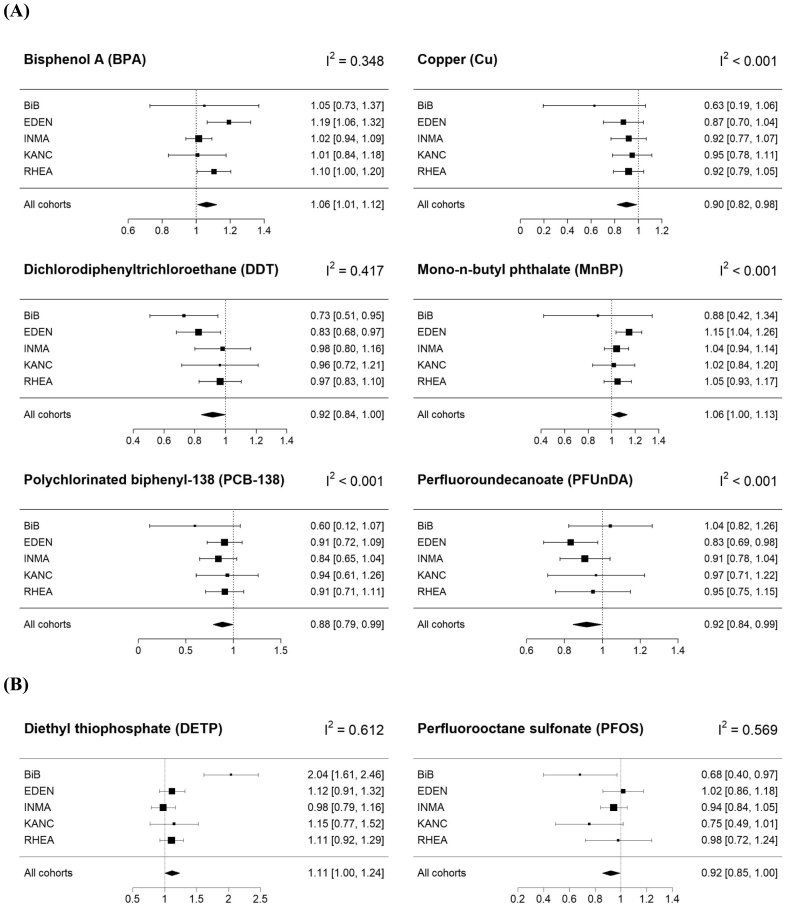
Sensitivity analysis (*n* = 708. BiB *n* = 46; EDEN *n* = 193; INMA *n* = 218; KANC *n* = 83; RHEA *n* = 168). Cohort-specific associations between prenatal exposures and SDQ externalizing (A) and internalizing (B) scores detected by the ExWAS (p value of association <0.05 except of diethyl thiophosphate for which the p value = 0.053). Regression models were adjusted for cohort, season of conception, child sex and age at SDQ assessment, parity, maternal: education level, work status, age, pre-pregnancy BMI and prenatal active smoking status. The “All cohorts” estimates are those obtained in the main ExWAS. IRRs are reported with 95%CIs and correspond to the change in the probability of the SDQ scores increasing by one unit for an IQR change in the log_2_ of the biomarker concentration in maternal blood or urine. We relied on the following threshold for I^2^ interpretation: I^2^ < 0.3 low heterogeneity, 0.3 ≤ I^2^ < 0.6 moderate heterogeneity, I^2^ ≥ 0.6 substantial to high heterogeneity. The black squares display the IRRs (size of the square reflects the relative size of each cohort) and the horizontal lines their 95%CIs. Abbreviations: BiB = Born in Bradford. EDEN = Étude des Déterminants Pré et Postnatals du Développement et de la Santé de l'Enfant. INMA = Infancia y Medio Ambiente. KANC = Kaunas Cohort. RHEA = Mother-Child Cohort in Crete. CI = confidence interval of the IRR estimate. ExWAS = exposome-wide association study. IQR = inter-quartile range. IRR = incidence rate ratio. BMI = body mass index. SDQ = Strengths and Difficulties Questionnaire.

**Fig. 2:**
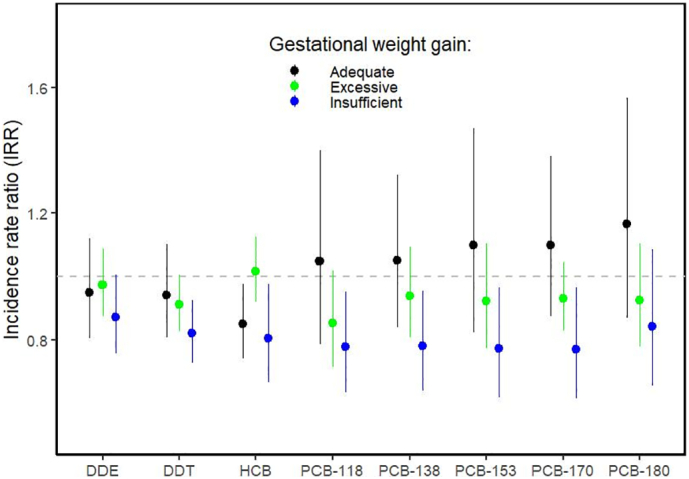
Sensitivity analysis for exposure-SDQ externalizing score associations stratified on gestational weight gain. We stratified on three categories of gestational weight gain as defined in the revised recommendations of the US Institute of Medicine (Institute of Medicine (US) and National Research Council (US) Committee to Reexamine IOM Pregnancy Weight Guidelines 2009): adequate (in black), excessive (in green) and insufficient (in blue). For pre-pregnancy BMI < 18.5 kg/m^2^ recommended total weight gain was 12.5–18.0 kg, for BMI = 18.5–24.9 kg/m^2^: 11.5–16.0 kg, for BMI = 25.0–29.9 kg/m^2^: 7.0–11.5 kg and for BMI ≥ 30.0 kg/m^2^ recommended weight gain was 5.0–9.0 kg. We ran one negative binomial regression model per exposure and outcome for each sub-population. IRRs are reported with 95%CIs and correspond to the change in the probability of the SDQ scores increasing by one unit for an IQR change in the log_2_ of the biomarker concentration in maternal blood. Each point represents the IRR estimate and the vertical line its 95%CI. Regression models were adjusted for cohort, season of conception, child sex and age at SDQ assessment, parity, and maternal factors: education level, work status, age and prenatal active smoking status. Abbreviations: BMI = body mass index. CI = confidence interval of the IRR estimate. IQR = inter-quartile range. IRR = incidence rate ratio. SDQ = Strengths and Difficulties Questionnaire. DDE = dichlorodiphenyldichloroethylene. DDT = dichlorodiphenyltrichloroethane. HCB = hexachlorobenzene. PCB = polychlorinated biphenyl. (For interpretation of the references to colour in this figure legend, the reader is referred to the web version of this article.)

After correction for multiple testing (corrected p value of 0.0017), none of the exposure-SDQ score associations passed the significance threshold. All exposure-SDQ score associations detected in our main analysis were linear (Appendix Fig. 2). Compared to the main ExWAS where each exposure biomarker was studied separately, adjustment for coexposures (i.e., exposures associated with the SDQ scores with a p value below 0.2) led to similar effect estimates except for PCB-138 (IRR: 0.95, 95%CI: 0.82;1.11) and PFOS (IRR: 0.94, 95%CI: 0.81;1.08) for which the negative association with SDQ scores was attenuated and the confidence intervals widened (Table 2). Effect estimates for analyses additionally adjusted for fish and seafood consumption (not shown) and breastfeeding (not shown) were similar to those observed in the main analysis. For the complete case analysis, while the effect estimates were similar to those of our main analysis, their confidence intervals were wider and the p values increased (e.g., p value = 0.212 and 0.141 for PFUnDA and DETP, respectively), likely because of the smaller sample size (n ranged from 314 for Cu to 646 for PFOS). No interactions with child's sex were detected for the associations highlighted in our main analysis (lowest p value for an interaction was 0.28 for DDT). Exclusion of the BiB cohort (new sample size *n* = 662, Table 2) did not strongly affect our results for the SDQ externalizing score, except of widening of the confidence interval for DDT (IRR: 0.92, 95%CI: 0.84;1.00 for the main ExWAS and IRR: 0.94, 95%CI: 0.86;1.03 after BiB cohort exclusion). We observed low heterogeneity across cohorts (I^2^ < 0.001) for most exposure-SDQ score associations (Fig. 1, Appendix Table 6, Appendix Table 7), except for BPA (I^2^ = 0.348, association mainly observed in EDEN and RHEA cohorts), DDT (I^2^ = 0.417, association mainly observed in BiB and EDEN cohorts), DETP (I^2^ = 0.612) and PFOS (I^2^ = 0.569). For PFOS, the association was driven by BiB and KANC, the two cohorts with the smallest sample size (*n* = 46 and 83 for BiB and KANC, respectively) and the lowest median value for PFOS concentration. Similarly, the association between DETP and SDQ internalizing score was mainly observed in BiB, the cohort with the smallest sample size. For the lipophilic compounds from the OCs family, stratification for gestational weight gain showed that the negative association was mainly observed among women with insufficient weight gain (Fig. 2).

## 4 Discussion

Among the 47 exposures tested, only seven were associated (uncorrected p values <0.05) with either externalizing or internalizing SDQ score in children between three and seven years of age. Association with one additional exposure biomarker was close to significance (uncorrected p value = 0.053). Cautious interpretation of the results is required since none passed the significance threshold after the FWER correction of the p values obtained in the ExWAS. For this reason, in the discussion we focused on the associations that were detected by both the LASSO and the ExWAS or that were consistent with previous human literature. The other associations should be treated as hypothesis generating.

Cu was detected by LASSO and by ExWAS as negatively associated with SDQ externalizing score, suggesting lower risk of behavioural problems. Cu is essential for many biological processes, including brain development during the foetal period (Scheiber et al., 2014), and an excess or insufficiency of Cu may lead to health problems (Gaetke et al., 2014). Our finding of a negative association between Cu and SDQ score (suggesting decreased risk of behavioural problems) needs to be replicated as, to our knowledge, the only study that assessed prenatal Cu and externalizing behaviour relied on the older children of the HELIX cohort (6–11 years) and did not report any effect (IRR: 1.00, 95%CI: 0.91;1.09, (Maitre et al. submitted to journal)). Cu concentrations in our study population (geometric mean = 1440 μg/L of blood, 95%CI: 1410;1471) were slightly higher than those reported among non-pregnant females in the most recent US NHANES study (geometric mean = 1270 μg/L, 95%CI: 1240;1300, Centers for Disease Control and Prevention, 2019). However, this may be due to the fact that serum Cu concentrations tend to increase during pregnancy (Vukelic et al., 2012).

Prenatal BPA urinary concentration was associated with higher (worse) scores on the externalizing behaviour sub-scale. Such association has also been suggested by another study assessing behaviour at older age on a similar population (IRR: 1.07, 95%CI: 0.99;1.16, Maitre et al. submitted to journal), suggesting that the association we observed between three and seven years of age might persist when the children get older. Previous studies coherently reported positive associations between prenatal BPA and externalizing behaviour scores (Braun et al., 2009, Braun et al., 2017b; Evans et al., 2014; Li et al., 2020; Perera et al., 2012; Philippat et al., 2017; Roen et al., 2015; Stacy et al., 2017) or the hyperactivity-inattention score (Casas et al., 2015), an item included in our externalizing SDQ score sub-scale. All the mentioned studies, except for those relying on the HOME mother-child cohort (Braun et al., 2009, Braun et al., 2017b; Stacy et al., 2017), reported these associations among boys, while we did not observe a sex-specific effect. Previous studies also reported higher scores on the internalizing behaviour sub-scale in association with the prenatal exposure to bisphenol A (Braun et al., 2011, Braun et al., 2017a; Evans et al., 2014; Grohs et al., 2019; Harley et al., 2013; Li et al., 2020; Perera et al., 2012, Perera et al., 2016; Philippat et al., 2017; Roen et al., 2015). While not significant (p value = 0.21), effect estimate for our study population also suggested a positive association between BPA and internalizing SDQ score (IRR: 1.04, 95%CI: 0.98;1.12). The animal research literature is also consistent here: numerous studies in rodents have reported a link between exposure to BPA and behaviour (Anderson et al., 2013; Ishido et al., 2011; Komada et al., 2014; Nakagami et al., 2009; Palanza et al., 2008; Rochester et al., 2018; Tian et al., 2010). Moreover, *in vitro* and *in vivo* studies provide evidence that BPA can affect biological pathways crucial for normal brain development by binding oestrogen receptors or interacting with the thyroid hormone and hypothalamic-pituitary-adrenal axis (Mustieles et al., 2015; Mustieles and Fernández, 2020; Nesan et al., 2018).

Maternal urinary MnBP concentration was associated with worse externalizing behaviour score. MnBP is a metabolite of dibutyl phthalate (DBP), a compound that exerts anti-androgenic activity (National Academies of Sciences, Engineering, and Medicine, 2017). Two previous human studies of prenatal MnBP concentration and child behaviour reported an association with externalizing behaviour among boys (Engel et al., 2010; Lien et al., 2015) and one reported an association with conduct problems, an item included in our externalizing behaviour sub-scale (Kobrosly et al., 2014). Other studies reported associations with other components of child behaviour (i.e., internalizing behaviour, Philippat et al., 2017; Whyatt et al., 2012) or no association for this phthalate metabolite (Engel et al., 2018; Gascon et al., 2015; Minatoya et al., 2018). Experimental studies in rodents also support a behavioural effect of MnBP (Farzanehfar et al., 2016; Hoshi and Ohtsuka, 2009; Yan et al., 2016). The heterogeneity of the epidemiological literature supports further investigation of the potential effect of MnBP on child behaviour.

DETP, a nonspecific dialkyl phosphate (DAP) metabolite, was the only OP pesticide metabolite associated with worse internalizing behaviour score. This association was on the verge of significance and showed a substantial heterogeneity between the cohorts. Moreover, maternal DETP concentration was the lowest among the OP pesticide metabolite family. OP pesticides are neurotoxic and there are several studies pointing towards the deleterious associations between prenatal concentrations of their metabolites and neurodevelopment in humans (reviewed in Sapbamrer and Hongsibsong, 2019; and Tessari et al., 2020). Nevertheless, few epidemiological studies have explored their potential effects on child behaviour. Results for the CHAMACOS cohort relying on the Child Behaviour Checklist suggested that the molar sum of DAP metabolites was associated with attention problems at five years (Marks et al., 2010) but not at earlier age (Eskenazi et al., 2007, Eskenazi et al., 2010). Another study found no link between DETP prenatal exposure and child behaviour (van den Dries et al., 2019). Our study is among the first ones to report the effect of prenatal exposure to DETP on child behaviour and, since the result was driven by the cohort with the smallest sample size (BiB, *n* = 46), it needs to be replicated.

DDT and PCB-138 were associated with lower SDQ externalizing scores, suggesting a protective effect on behaviour. Our sensitivity analysis showed that these protective effects were mainly seen among women with insufficient weight gain during pregnancy. Similar patterns of associations were observed for other compounds from the OCs family. Excessive gestational weight gain has been associated with both lower blood concentrations of lipophilic compounds (such as OCs) due to their storage in fat tissue (Lee et al., 2017), and with higher risk of behavioural problems in children (Pugh et al., 2016). However, since previous studies did not report protective effects for DDT and PCBs on child behaviour (Forns et al., 2016; Rosenquist et al., 2017) the associations we observed between OCs and SDQ scores should be interpreted with caution.

We found a negative association between two PFASs (PFOS and PFUnDA) and child behaviour. The association with PFOS was only observed in the two cohorts with the smallest sample size (BiB and KANC with *n* = 46 and 83, respectively) and the lowest median PFOS concentrations. The association with PFOS was not expected as previous human studies reported either increased behavioural problems linked to this exposure (Høyer et al., 2015) or no association at all (Fei and Olsen, 2011; Forns et al., 2015). The negative association between PFUnDA and child behaviour was also observed among older children of the HELIX cohorts (IRR: 0.89, 95%CI: 0.80;0.98, Maitre et al. submitted to journal) and needs further investigation.

## 5 Strengths and limitations

Our study is among the first to simultaneously consider a large number of exposures (*n* = 47) from multiple families in relation to externalizing and internalizing behaviour scores in children. Its strengths include the longitudinal design, which allows prospectively assessing exposure during pregnancy (a critical period for brain development), and the use of a standardized tool (SDQ) to evaluate child behaviour. We relied on two complementary statistical approaches: ExWAS produces effect estimates that are comparable to previous studies and can be used in meta-analyses, while LASSO considers all exposures simultaneously, performs variable selection, and is on average less likely to generate false positives (spurious associations) than ExWAS (Barrera-Gómez et al., 2017). Moreover, we investigated potential coexposure confounding: the associations for BPA, Cu, DDT, DETP, MnBP and PFUnDA remained after adjusting for other exposures. Finally, relying on five cohorts with differing confounding structure (e.g., women from the BiB cohort had overall lower education levels) might improve causal inference: an association seen in multiple heterogeneous cohorts is less likely to result from residual confounding than an association seen in only one or a few homogenous cohorts (Richmond et al., 2014). On the other hand, since the cohorts were recruited before the start of the HELIX project, collection of biological samples during pregnancy was not harmonized leading to different timings (i.e., different trimester) for exposure assessment across cohorts. Additionally, for some cohorts the same exposure biomarker was sometimes assessed by different laboratories (see Appendix Table 2), which may partly explain the between-cohort heterogeneity of the results observed for some exposures. This should not have a strong impact on our results since the interlaboratory comparisons performed in the framework of the HELIX protocol suggested a high correlation between assessments performed in different laboratories. For instance, correlation coefficients between phenol urinary concentrations measured by the Norwegian Institute of Public Health and Centers for Disease Control and Prevention in 12 maternal samples of the EDEN cohort were ≥ 0.90 (Supplementary of Tamayo-Uria et al., 2019). Due to limited availability of biological samples, some biomarkers of exposure were not assessed in all cohorts (e.g., metals and semi-metals were not assessed in INMA, see Appendix Table 5 for details). We used multiple imputation on the missing values as it has been shown to generate less bias than exclusion of a variable or a stratum (e.g., exclusion of an entire cohort for which an exposure biomarker concentration was fully missing, Held et al., 2016; Jolani et al., 2015). Nevertheless, for the exposures with many missing values (metals, PBDEs) multiple imputation may have widened the confidence intervals of our effect estimates, limiting the ability to detect associations. Moreover, we relied on spot urine samples to assess exposure to compounds whose urinary concentration has moderate to high intra-individual variability during pregnancy (reviewed by Casas et al., 2018). This can potentially lead to exposure misclassification, attenuation bias and power reduction (Perrier et al., 2016). It has been shown that the measurement error and resulting attenuation of the effect estimates varies across exposures (exposures with the most intra-individual variability have the highest errors, Perrier et al., 2016). Therefore, we must be cautious when comparing exposure-SDQ associations across exposures with differing intra-individual variability. We decided not to assess all possible second-order interactions between exposures because, given our limited sample size and large number of exposures, it could have substantially decreased the power and increased the false positive rate (Barrera-Gómez et al., 2017). Finally, we focused only on prenatal exposure and did not assess exposure in early postnatal life, a period also recognized as crucial for brain development.

## 6 Conclusion

In line with previous epidemiological studies, our results suggested a deleterious association between prenatal exposure to bisphenol A, MnBP (a metabolite of DBP) and child behaviour. According to the toxicological literature, the association observed for BPA is biologically plausible. DETP was also associated with worse behavioural scores, however this result should be interpreted with caution since it was driven by the smallest cohort. Cu, DDT, PCB-138, PFOS and PFUnDA were associated with lower risk of behavioural problems. These associations were not reported previously and for lipophilic compounds (DDT and PCB-138) could even result from changes in body composition during pregnancy.

## Funding/Support

P. Jedynak was funded by the 10.13039/501100001665French National Research Agency in the framework of the Investissements d'Avenir program (ANR-15-IDEX-02). J. Julvez holds the Miguel Servet-II contract (CPII19/00015) awarded by the Instituto de Salud Carlos III (co-funded by the European Social Fund “Investing in your future”). The study has received funding from the European Community's 10.13039/100011102Seventh Framework Programme (FP7/2007–2013) under grant agreement no 308333 for the HELIX project. The present work relied on data from five out of six HELIX cohorts that received funding previously. INMA study data collection was supported by grants from the Instituto de Salud Carlos III, CIBERESP and the Generalitat de Catalunya-CIRIT (Spain). KANC study was funded by the grant of the Lithuanian Agency for Science Innovation and Technology (6-04-2014-31V-66). The RHEA study was financially supported by European projects and the Greek Ministry of Health and Social Solidarity (Program of Prevention of obesity and neurodevelopmental disorders in preschool children, in Heraklion district, Crete, Greece: 2011–2014; “Rhea Plus”: Primary Prevention Program of Environmental Risk Factors for Reproductive Health and Child Health: 2012–2015). Core support for BiB study was provided by the Wellcome Trust (WT101597MA, UK). EDEN study was supported by grants from Foundation for Medical Research (FRM), Inserm, French Institute for Public Health Research (IReSP), Nestlé, French Ministry of Social Affairs and Health, French National Research Agency (ANR), Université Paris-Sud, French Institute for Public Health Surveillance (InVS), French Agency for Food, Environmental and Occupational Health & Safety (ANSES) and Mutuelle générale de l'Education nationale (MGEN).

## Role of the funder/sponsor

The funding sources had no role in any of: the design and conduct of the study; collection, management, analysis, and interpretation of the data; preparation, review or approval of the manuscript; decision to submit the manuscript for publication.

## CRediT authorship contribution statement

Jedynak had full access to all the data in the study and takes responsibility for the integrity of the data and the accuracy of the data analysis.

Study concept and design: Vrijheid.

Supervision of all aspects of study design and data collection: Vrijheid.

Cohort data collection: Gützkow, Guxens, Casas, McEachan, Gražulevičienė, Chatzi, Kampouri, Vafeiadi, Vrijheid, Slama, Mon-Williams, Wright.

Acquisition, analysis, or interpretation of data: Jedynak, Philippat, Julvez, López-Vicente, Tamayo, Sunyer, Casas, Thomsen, Vrijheid, Maitre.

Drafting of the manuscript: Jedynak, Philippat.

Statistical analysis: Jedynak, Basagaña, Philippat.

Obtained funding: Thomsen, Slama, Chatzi, Wright, Gražulevičienė, Vrijheid.

Technical or material support: Urquiza.

Study supervision: Philippat.

All authors have read, commented on and approved the manuscript.

## Declaration of competing interest

The authors declare that they have no known competing financial interests or personal relationships that could have appeared to influence the work reported in this paper.

## Appendix A Supplementary data

Supplementary material Image 1
