# Supplementary material for: Prenatal exposure to a wide range of environmental chemicals and child behaviour between 3 and 7 years of age – An exposome-based approach in 5 European cohorts
Source: Sci Total Environ. 2021 Apr 1;763:144115. doi: 10.1016/j.scitotenv.2020.144115 (PMC7840589; doi:10.1016/j.scitotenv.2020.144115)
Supplement: Supplementary file 1 — Supplementary material [file mmc1.docx]

#### Supplementary materials

**Prenatal exposure to a wide range of environmental chemicals and child behaviour between 3 to 7 years – an exposome-based approach in 5 European cohorts**

Paulina Jedynak, Léa Maitre, Mónica Guxens, Kristine B. Gützkow, Jordi Julvez, Mónica López-Vicente, Jordi Sunyer, Maribel Casas, Leda Chatzi, Regina Gražulevičienė, Mariza Kampouri, Rosie McEachan, Mark Mon-Williams, Ibon Tamayo, Cathrine Thomsen, José Urquiza, Marina Vafeiadi, John Wright, Xavier Basagaña, Martine Vrijheid, Claire Philippat

#### List of Appendix Figures

**Appendix Figure 1:** Study flow-chart. 3

**Appendix Figure 2:** GAMs with restricted cubic splines function fitted on the prenatal concentrations of exposures selected by the LASSO and ExWAS as associated with the SDQ score. 4

#### List of Appendix Tables

**Appendix Table 1:** List of prenatal exposures assessed in this study. 3

**Appendix Table 2:** Exposure assessment – biological matrices, timing of sample collection, laboratories, methodologies and quality controls 6

**Appendix Table 3:** Scoring of the Strengths and Difficulties Questionnaire. 10

**Appendix Table 4:** Adjustment factors. 11

**Appendix Table 5:** Descriptive statistics of all prenatal exposures: overall and by cohort. 12

**Appendix Table 6:** Adjusted associations between prenatal exposure to environmental contaminants and SDQ *externalising* score. 14

**Appendix Table 7:** Adjusted associations between prenatal exposure to environmental contaminants and SDQ *internalising* score. 16

#### Appendix Figures and Tables


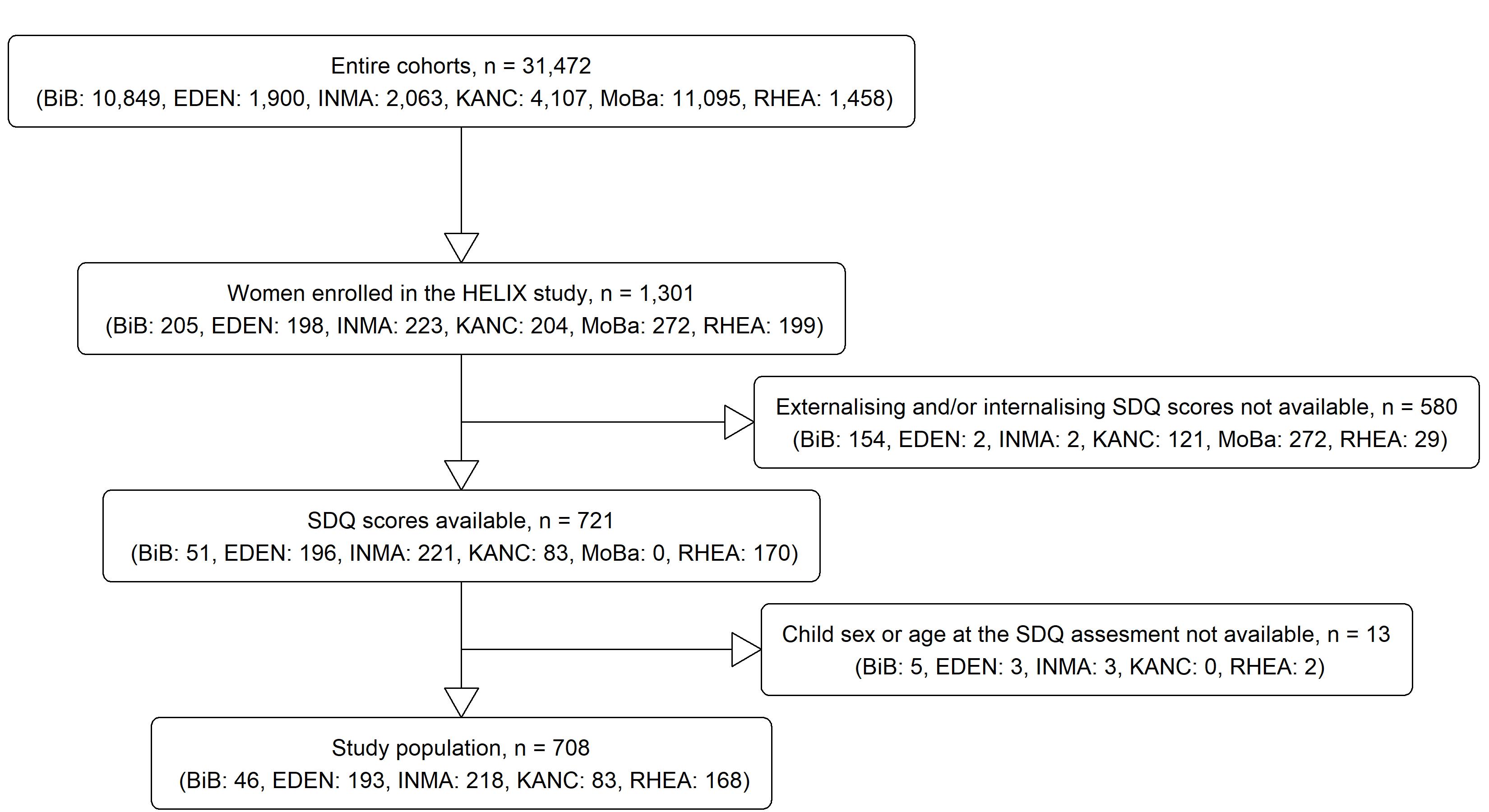


**Appendix Figure 1:** Study flow-chart. Abbreviations: BiB = Born in Bradford. EDEN = Étude des Déterminants Pré et Postnatals du Développement et de la Santé de l’Enfant. INMA = Infancia y Medio Ambiente. KANC = Kaunas Cohort. MoBa = Norwegian Mother, Father and Child Cohort Study. RHEA = Mother-Child Cohort in Crete. SDQ = Strengths and Difficulties Questionnaire. Adapted from Maitre et al. 2018.

**(A)**


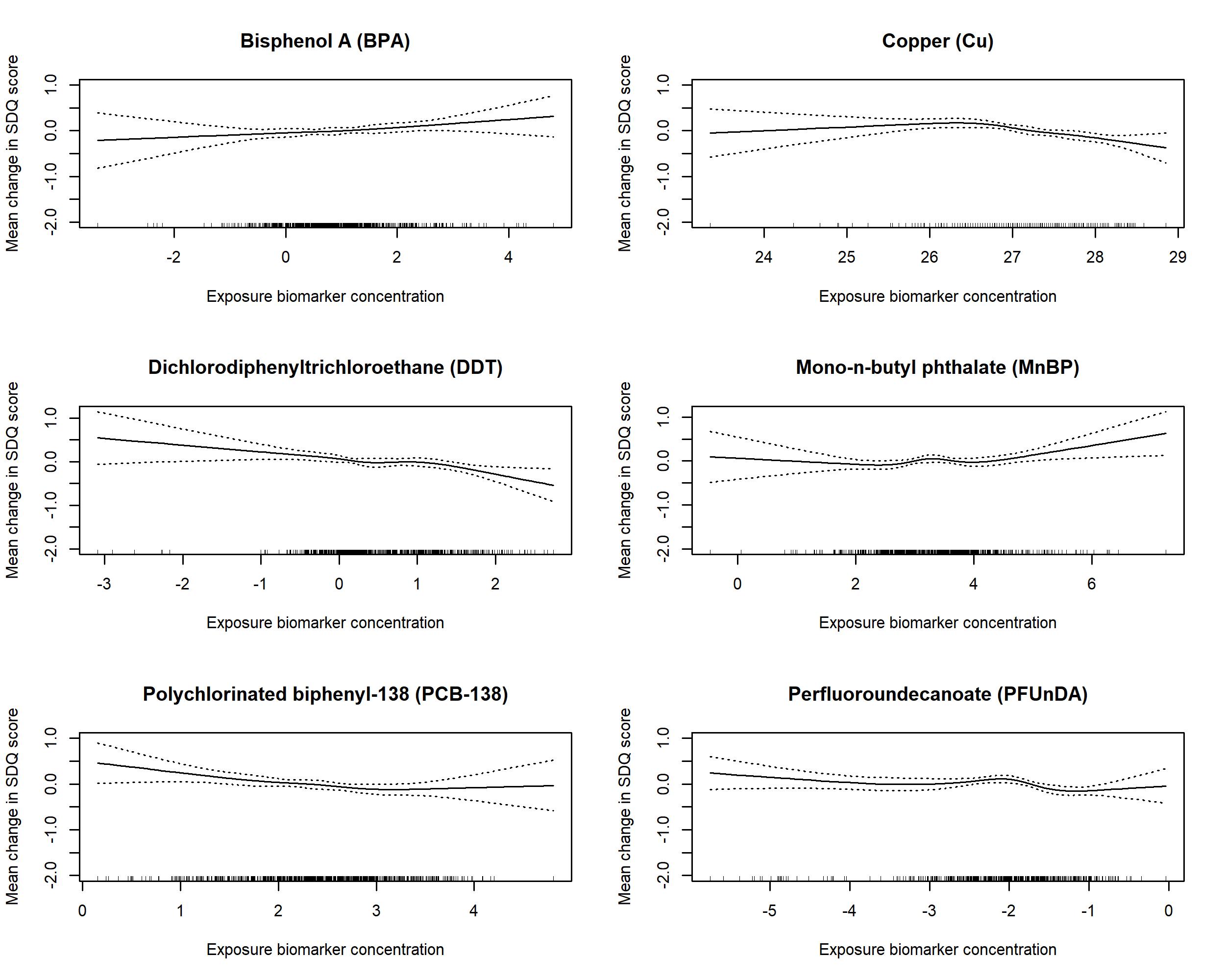


**(B)**

***
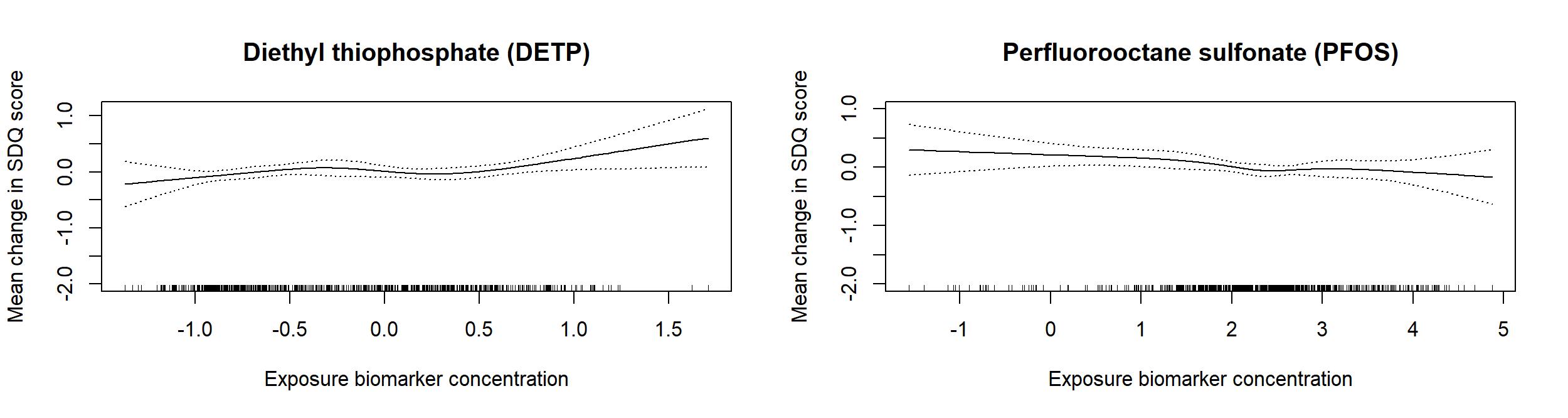
***

**Appendix Figure 2:** GAMs with restricted cubic splines function fitted on the log_2_ and IQR transformed prenatal concentrations of exposures selected by the LASSO and ExWAS as associated with the externalising (A) and internalising (B) SDQ scores. Solid line represents the fit and dashed line the standard error of the fit. Models were adjusted for cohort, season of conception, child sex and age at SDQ assessment, parity, and maternal factors: education level, work status, age, pre- pregnancy BMI, and prenatal active smoking status. Abbreviations: GAM = generalized additive model. ExWAS = exposome-wide association study. IQR = inter-quartile range. LASSO = least absolute shrinkage and selection operator. SDQ = Strengths and Difficulties Questionnaire.

**Appendix Table 1:** List of prenatal exposures assessed in this study.

| **Exposure family** | **Unit and biological matrix** | **Description of exposures** |
| --- | --- | --- |
| Organochlorine compounds (OCs) | ng/g of lipids in serum or plasma | Dichlorodiphenyldichloroethylene (DDE), dichlorodiphenyltrichloroethane (DDT), hexachlorobenzene (HCB), polychlorinated biphenyls (PCBs): PCB-118, PCB-138, PCB-153, PCB-170, PCB-180 |
| Polybrominated diphenyl ethers (PBDEs) | ng/g of lipids in serum or plasma | Polybrominated diphenyl ethers (PBDEs): PBDE-47, PBDE-153 |
| Per- and polyfluoroalkyl substances (PFASs) | µg/L in serum or plasma or whole blood | Perfluorohexane sulfonate (PFHxS), perfluorononanoate (PFNA), perfluorooctanoate (PFOA), perfluorooctane sulfonate (PFOS), perfluoroundecanoate (PFUnDA) |
| Metals and semi-metals (essential and toxic elements) | µg/L of whole blood or cord blood | Inorganic arsenic (As), cadmium (Cd), caesium (Cs), cobalt (Co), copper (Cu), lead (Pb), manganese (Mn), mercury (Hg), molybdenum (Mo) |
| Phthalate metabolites | µg/g of creatinine in urine | Mono benzyl phthalate (MBzP), mono-2-ethyl 5-carboxypentyl phthalate (MECPP), mono-2-ethyl-5-hydroxyhexyl phthalate (MEHHP), mono-2-ethylhexyl phthalate (MEHP), mono-2-ethyl-5-oxohexyl phthalate (MEOHP), monoethyl phthalate (MEP), mono-iso-butyl phthalate (MiBP), mono-n-butyl phthalate (MnBP), mono-4-methyl-7-hydroxyoctyl phthalate (ohMiNP), mono-4-methyl-7-oxooctyl phthalate (oxo-MiNP) |
| Phenols | µg/g of creatinine in urine | Bisphenol A (BPA), n-butyl paraben (BUPA), ethyl paraben (ETPA), methyl paraben (MEPA), oxybenzone (OXBE), propyl paraben (PRPA), triclosan (TRCS) |
| Organophosphate (OP) pesticide metabolites | µg/g of creatinine in urine | Diethyl phosphate (DEP), diethyl thiophosphate (DETP), dimethyl dithiophosphate (DMDTP), dimethyl phosphate (DMP), dimethyl thiophosphate (DMTP) |
| Marker of smoking | µg/g of creatinine in urine | Cotinine |

**Appendix Table 2:** Exposure assessment – biological matrices, timing of sample collection, laboratories, methodologies and quality controls (adapted from Haug et al. 2018).

|  |  |  | **Cohort** |  |  |
| --- | --- | --- | --- | --- | --- |
| **Family of exposures** | **BiB** | **EDEN** | **KANC** | **INMA** | **RHEA** |
| **OCs** |  |  |  |  |  |
| Biological matrix | Serum/plasma | Serum | - | Serum | Serum |
| Timing of sample collection, mean GW (SD) | 26.6 (1.4) | 26.1 (1.2) | 13.7 (2.0) | 39.4 (1.3) | 14.1 (3.7) |
| Laboratory | NIPH (Norway) | NIPH (Norway) | NIPH (Norway) | LSPG (Spain) | National Institute for Health and Welfare, Chemical Exposure Unit, Kuopio (Finland) |
| Analytical method | GC-MS/MS | GC-MS/MS | GC-MS/MS | GC-MS | GC-MS/MS |
| Interlaboratory comparison | We participated 3 times in the AMAP interlaboratory comparison study (Arctic Monitoring and Assessment Program “Ring Test for Persistent Organic Pollutants in Human Serum) during the period when HELIX samples were analysed. Each round included 3 samples. The samples are spiked, and most analytes are in a much higher concentration range than our samples usually are. Despite these factors Z- scores below 2 were obtained for all contaminants except for PCB 153, and for PCB 170 in one sample. | | | | |
| Standard reference material | Two different SRMs were obtained from NIST (National Institute of Standards and Technology, USA), Organic Contaminants in Human Serum, non-fortified (1957) and fortified (1958). The RSDs of the 6 different injections of the non-fortified sample (1957) were 2-18%. The deviation from the certified values was 2-20%, except for HCB (50%) and PCB-153 (76%), where a small blank contribution may be the problem. The RSDs of the 6 different injections of the fortified sample (1958) were 2-20%, and for most analytes around 5%. The deviation from the certified values were 2-17%, except for DDT (-40%). | | | | |
| Reference for the analytic method | Modified (Caspersen et al. 2016) | Modified (Caspersen et al. 2016) | Modified (Caspersen et al. 2016) | (Goñi et al. 2007) | (Koponen et al. 2013) |
| **PBDEs** |  |  |  |  |  |
| Biological matrix | Serum/plasma | Serum | - | Serum | Serum |
| Timing of sample collection, mean GW (SD) | 26.6 (1.4) | 26.1 (1.2) | 13.7 (2.0) | 39.4 (1.3) | 14.1 (3.7) |
| Laboratory | NIPH (Norway) | NIPH (Norway) | NIPH (Norway) | NIPH (Norway) | National Institute for Health and Welfare, Chemical Exposure Unit, Kuopio (Finland) |
| Analytical method | GC-MS/MS | GC-MS/MS | GC-MS/MS | GC-MS | GC-MS/MS |
| Interlaboratory comparison | We participated 3 times in the AMAP interlaboratory comparison study (Arctic Monitoring and Assessment Program “Ring Test for Persistent Organic Pollutants in Human Serum) during the period when HELIX samples were analysed. Each round included 3 samples. The samples are spiked, and most analytes are in a much higher concentration range than our samples usually are. Despite these factors Z- scores below 2 were obtained for all contaminants. | | | | |
| Standard reference material | Two different SRMs were obtained from NIST (National Institute of Standards and Technology, USA), Organic Contaminants in Human Serum, non-fortified (1957) and fortified (1958). The RSDs of the 6 different injections of the non-fortified sample (1957) were 2-18%. The deviation from the certified values was 2-20%. The RSDs of the 6 different injections of the fortified sample (1958) were 2-20%, and for most analytes around 5%. The deviation from the certified values were 2-17%. | | | | |
| Reference for the analytic method | Modified (Caspersen et al. 2016) | Modified (Caspersen et al. 2016) | Modified (Caspersen et al. 2016) | Modified (Caspersen et al. 2016) | (Koponen et al. 2013) |
| **PFASs** |  |  |  |  |  |
| Biological matrix | Serum/plasma | Serum | Whole blood | Plasma | Serum |
| Timing of sample collection, mean GW (SD) | 26.6 (1.4) | 26.1 (1.2) | 13.7 (2.0) | 39.4 (1.3) | 14.1 (3.7) |
| Laboratory | NIPH (Norway) | NIPH (Norway) | NIPH (Norway) | Institute for Occupational Medicine, RWTH Aachen University (Germany) | NIPH (Norway) |
| Analytical method | GC-MS | GC-MS | GC-MS | Online column-switching LC–MS–MS analysis | GC-MS |
| Interlaboratory comparison | We participated 3 times in the AMAP interlaboratory comparison study (Arctic Monitoring and Assessment Program “Ring Test for Persistent Organic Pollutants in Human Serum) during the period where HELIX samples were analysed. Each round included 3 samples. The samples are spiked in a wide concentration range. Z- scores below 2 were obtained for all contaminants except for PFUnDA in two samples. | | | | |
| Standard reference material | In total 15 samples from 4 rounds of the AMAP interlaboratory comparison study (Arctic Monitoring and Assessment Program “Ring Test for Persistent Organic Pollutants in Human Serum) were analysed during the period where HELIX samples were analysed, and the mean deviation from the assigned value varied between 8 and 17%. | | | | |
| Reference for the analytic method | (Haug et al. 2009) | (Haug et al. 2009) | (Poothong et al. 2017) | (Manzano-Salgado et al. 2015) | (Haug et al. 2009) |
| **Metals and semi-metals** |  |  |  |  |  |
| Biological matrix | Whole blood | Whole blood | Whole blood | Cord whole blood | Whole blood |
| Timing of sample collection, mean GW (SD) | 26.6 (1.4) | 26.1 (1.2) | 13.7 (2.0) | 39.4 (1.3) | 14.1 (3.7) |
| Laboratory | ALS Scandinavia (Sweden) | ALS Scandinavia (Sweden) | ALS Scandinavia (Sweden) | Hg; LSPA (Spain) | ALS Scandinavia (Sweden) |
| Analytical method | ICP-SFMS | ICP-SFMS | ICP-SFMS | AAS | ICP-SFMS |
| Standard reference material | In total 15 samples of two Seronorm whole blood reference materials were provided to ALS Scandinavia which performed analyses of toxic and essential elements of the HELIX samples. The samples were blinded for the laboratory. The mean deviation from the assigned value varied between 1 and 17%, except for Co which was 53%. | | | | |
| Reference for the analytic method | (Rodushkin et al. 2000) | (Rodushkin et al. 2000) | (Rodushkin et al. 2000) | (Ramon et al. 2011) | (Rodushkin et al. 2000) |
| **Phthalate metabolites** |  |  |  |  |  |
| Biological matrix | Urine | Urine | - | Urine | Urine |
| Timing of sample collection, mean GW (SD) | 26.6 (1.4) | 26.1 (1.2) | 34.2 (1.3) | NA | 14.1 (3.7) |
| Laboratory | NIPH (Norway) | NIPH (Norway) | NIPH (Norway) | Bioanalysis Research Group at the Hospital del Mar Medical Research Institute (Spain) | NIPH (Norway) |
| Analytical method | LC-MS/MS | LC-MS/MS | LC-MS/MS | HPLC-MS | LC-MS/MS |
| Standard reference material | In total 42 samples of NIST reference material SRM 3673 were analysed during the period where HELIX samples were analysed, and the mean deviation from the assigned value varied between 1 and 25%. | | | | |
| Reference for the analytic method | (Sabaredzovic et al. 2015) | (Sabaredzovic et al. 2015) | (Sabaredzovic et al. 2015) | (Valvi et al. 2015) | (Sabaredzovic et al. 2015) |
| **Phenols** |  |  |  |  |  |
| Biological matrix | Urine | Urine | - | Urine | Urine |
| Timing of sample collection, mean GW (SD) | 26.6 (1.4) | 26.1 (1.2) | 34.2 (1.3) | NA | 14.1 (3.7) |
| Laboratory | NIPH (Norway) | National Center for Environmental Health laboratory at the CDC (US) | NIPH (Norway) | NIPH (Norway) | NIPH (Norway) |
| Analytical method | UPLC-MS/MS | Described in (Philippat et al. 2012) | UPLC-MS/MS | UPLC-MS/MS | UPLC-MS/MS |
| Interlaboratory comparison | We participated twice in the External Quality Assessment Scheme for organic substances in urine – OSEQAS, for BPA and TCS. Each round included 2 samples. Z- scores below 2 were obtained for both contaminants in all samples. | | | | |
| Standard reference material | In total 145 samples of NIST reference material SRM 3673 were analysed during the period where HELIX samples were analysed, and the mean deviation from the assigned value varied between 1 and 19%, except BPA which was 63%. | | | | |
| Reference for the analytic method | (Sakhi et al. 2018) | (Philippat et al. 2012) | (Sakhi et al. 2018) | (Sakhi et al. 2018) | (Sakhi et al. 2018) |
| **OP pesticide metabolites** |  |  |  |  |  |
| Biological matrix | Urine | Urine | - | Urine | Urine |
| Timing of sample collection, mean GW (SD) | 26.6 (1.4) | 26.1 (1.2) | 34.2 (1.3) | NA | 14.1 (3.7) |
| Laboratory | NIPH (Norway) | NIPH (Norway) | NIPH (Norway) | NIPH (Norway) | NIPH (Norway) |
| Analytical method | UPLC-TOF | UPLC-TOF | UPLC-TOF | UPLC-TOF | UPLC-TOF |
| Reference | (Cequier et al. 2016) | (Cequier et al. 2016) | (Cequier et al. 2016) | (Cequier et al. 2016) | (Cequier et al. 2016) |
| **Cotinine** |  |  |  |  |  |
| Biological matrix | Urine | Urine | - | Urine | Urine |
| Timing of sample collection, mean GW (SD) | 26.6 (1.4) | 26.1 (1.2) | 34.2 (1.3) | NA | 14.1 (3.7) |
| Laboratory | Fürst Medical Analysis Laboratory (Norway) | Fürst Medical Analysis Laboratory (Norway) | Fürst Medical Analysis Laboratory (Norway) | Public Health Laboratory of Bilbao - LSPPV (Spain) | Fürst Medical Analysis Laboratory (Norway) |
| Analytical method | The Immulite® 2000 Nicotine Metabolite (Cotinine) 600 Test on an Immulite 2000 XPi from Siemens Healthineers | The Immulite® 2000 Nicotine Metabolite (Cotinine) 600 Test on an Immulite 2000 XPi from Siemens Healthineers | The Immulite® 2000 Nicotine Metabolite (Cotinine) 600 Test on an Immulite 2000 XPi from Siemens Healthineers | LC-MS | The Immulite® 2000 Nicotine Metabolite (Cotinine) 600 Test on an Immulite 2000 XPi from Siemens Healthineers |
| Reference for the analytic method | - | - | - | (Aurrekoetxea et al. 2013) | - |
| **Creatinine** |  |  |  |  |  |
| Biological matrix | Urine | Urine | - | Urine | Urine |
| Timing of sample collection, mean GW (SD) | 26.6 (1.4) | 26.1 (1.2) | 34.2 (1.3) | NA | 14.1 (3.7) |
| Laboratory | Fürst Medical Analysis Laboratory (Norway) | National Center for Environmental Health laboratory at the CDC (US) | Fürst Medical Analysis Laboratory (Norway) | Public Health Echevarne Laboratory of Barcelona (Spain) | Fürst Medical Analysis Laboratory (Norway) |
| Analytical method | AU680 Chemistry System form Beckman Coulter using DRI® Creatinine-Detect® Test | Enzymatic reaction using a Roche Hitachi 912 chemistry analyzer (Roche Hitachi, Basel, Switzerland) | AU680 Chemistry System form Beckman Coulter using DRI® Creatinine-Detect® Test | Jaffé method - Beckman Coulter© AU5400 | AU680 Chemistry System form Beckman Coulter using DRI® Creatinine-Detect® Test |
| Reference for the analytic method | - | - | - | - | - |
| **Lipids** |  |  |  |  |  |
| Biological matrix | Serum/plasma | Serum | - | Serum | Serum |
| Timing of sample collection, mean GW (SD) | 26.6 (1.4) | 26.1 (1.2) | 13.7 (2.0) | 39.4 (1.3) | 14.1 (3.7) |
| Laboratory | Fürst Medical Analysis Laboratory (Norway) | Fürst Medical Analysis Laboratory (Norway) | Fürst Medical Analysis Laboratory (Norway) | Laboratorio de Salud Pública de Bizkaia (Spain) | Medicon Hellas SA, Gerakas (Greece) |
| Analytical method | ADVIA® Chemistry XPT System, and the FS kit from DiaSys was used to measure concentrations of phospholipids | ADVIA® Chemistry XPT System, and the FS kit from DiaSys was used to measure concentrations of phospholipids | ADVIA® Chemistry XPT System, and the FS kit from DiaSys was used to measure concentrations of phospholipids | Cobas Mira self-analyzer (Roche Diagnostic, Basel, Switzerland) using an Enzymatic-Colorimetric method with Spinreact reagents | Standard enzymatic method |
| Reference for the analytic method | - | - | - | - | - |

Abbreviations: GW = gestational week. NA = not available. SD = standard deviation. BPA = bisphenol A. DDT = dichlorodiphenyltrichloroethane. HCB = hexachlorobenzene. OC = organochlorine compound. OP = organophosphate. PBDE = polybrominated diphenyl ether. PCB = polychlorinated biphenyl. PFAS = per- and polyfluoroalkyl substance. PFUnDA = perfluoroundecanoate. TRCS = triclosan. RSD = relative standard deviation. SRM = Standard reference material. AAS = thermal decomposition, amalgamation and atomic absorption spectrometry. GC-MS/MS = gas chromatography coupled with tandem mass spectrometry. GC-MS = gas chromatography mass spectrometry. GC-MS-NICI = gas chromatography-negative-ion chemical ionization mass spectrometry. HPLC-MS = ultra-performance liquid chromatography coupled to tandem mass spectrometry. ICP-SFMS = conductively coupled plasma-sector field mass spectrometry. LC-MS/MS = liquid chromatography-mass spectrometry. Q-ICP-MS = inductively coupled plasma quadruple mass spectrometry. SPE-HPLC–MS/MS = on-line solid-phase extraction coupled to isotope dilution high performance liquid chromatography-tandem mass spectrometry. UPLC-MS/MS = ultra-performance liquid chromatography-tandem mass spectrometry. UPLC-TOF = ultra-performance liquid chromatography coupled to time-of-flight mass spectrometry. AMAP = Arctic Monitoring and Assessment Program “Ring Test for Persistent Organic Pollutants in Human Serum”. CDC = Centers for Disease Control and Prevention. LSPA = Laboratorio de Salud Pública de Alava. LSPG = Laboratorio de Salud Pública de Guipúzcoa. NIPH = Norwegian Institute of Public Health. NIST = National Institute of Standards and Technology, USA. BiB = Born in Bradford. EDEN = Étude des Déterminants Pré et Postnatals du Développement et de la Santé de l’Enfant. INMA = Infancia y Medio Ambiente. KANC = Kaunas Cohort. RHEA = Mother-Child Cohort in Crete.

**Appendix Table 3:** Scoring of the Strengths and Difficulties Questionnaire.

| **Clinical behaviours (score ranges: 0-20)** | **Sub-scale (score ranges: 0-10)** | **Items used to compute each sub-scale (score ranges: 0-2)** |
| --- | --- | --- |
|  |  |  |
| Externalizing behaviour | Conduct problems | Often has temper tantrums or hot tempers |
|  |  | Generally obedient |
|  |  | Often fights with other children |
|  |  | Often argumentative with adults |
|  |  | Can be spiteful to others |
|  | Hyperactivity / Inattention problems | Restless, overactive |
|  |  | Constantly fidgeting or squirming |
|  |  | Easily distracted, concentration wanders |
|  |  | Can stop and think things out before acting |
|  |  | Sees tasks through to the end |
| Internalizing behaviour | Emotional symptoms | Often complains of headaches |
|  |  | Many worries |
|  |  | Often unhappy, downhearted |
|  |  | Nervous or clingy in new situations |
|  |  | Many fears, easily scared |
|  | Peer relationship problems | Rather solitary, tends to play alone |
|  |  | Has at least one good friend |
|  |  | Generally liked by other children |
|  |  | Picked on or bullied |
|  |  | Gets on better with adults than with other children |
|  | Prosocial behaviour | Considerate of other people's feelings |
|  |  | Shares readily with other children |
|  |  | Helpful if someone is hurt |
|  |  | Kind to younger children |
|  |  | Often volunteers to help others |

**Appendix Table 4:** Adjustment factors.

| Adjustment factor | Coding |
| --- | --- |
| Cohort | BiB / EDEN / INMA / KANC / RHEA |
| Season of conception | January-March / April-June / July-September / October-December |
| Active smoking during pregnancy | No / yes |
| Parity | Nulliparous / 1 child / ≥ 2 children |
| Maternal level of education | Primary school / secondary school / university degree or higher |
| Maternal work status during pregnancy | Unemployed / employed |
| Maternal pre-pregnancy BMI^a^ | Underweight (< 18.5 kg/m^2^) / normal weight (≥ 18 - < 25 kg/m^2^) / overweight (≥ 25 - < 30 kg/m^2^) / obesity (≥ 30 kg/m^2^) |
| Child sex | Female / male |
| Child age at the SDQ assessment | Continuous |
| Maternal age | Continuous |

**^a^** Categorized according to the World Health Organization definitions. Abbreviations: BiB = Born in Bradford. EDEN = Étude des Déterminants Pré et Postnatals du Développement et de la Santé de l’Enfant. INMA = Infancia y Medio Ambiente. KANC = Kaunas Cohort. RHEA = Mother-Child Cohort in Crete. BMI = body mass index. SDQ = Strengths and Difficulties Questionnaire.

**Appendix Table 5:** Descriptive statistics of all prenatal exposures: overall and by cohort.

| **Exposure family** | **Exposure** | **Missing: Nb. (%)** | **> LOD: Nb. (%)** | **Median (IQR)** | | | | | | **p value of equality between cohorts^a^** |
| --- | --- | --- | --- | --- | --- | --- | --- | --- | --- | --- |
|  |  |  |  | **Overall** | **BiB** | **EDEN** | **INMA** | **KANC** | **RHEA** |  |
| Metals and semi-metals (essential and toxic elements) | Inorganic arsenic (As) | 373 (52.7) | 179 (53.4) | 1.1 (1.7) | 1.4 (1.6) | 1.4 (2.3) | NA | 0.3 (0.0) | 1.5 (2.1) | < 0.0001 |
|  | Cadmium (Cd) | 373 (52.7) | 334 (99.7) | 0.2 (0.2) | 0.2 (0.1) | 0.2 (0.2) | NA | 0.2 (0.1) | 0.3 (0.2) | < 0.0001 |
|  | Cobalt (Co) | 373 (52.7) | 335 (100.0) | 0.2 (0.2) | 0.2 (0.5) | 0.2 (0.2) | NA | 0.2 (0.1) | 0.2 (0.2) | 0.139 |
|  | Caesium (Cs) | 373 (52.7) | 335 (100.0) | 1.4 (0.7) | 1.1 (0.5) | 1.9 (1.0) | NA | 1.1 (0.5) | 1.5 (0.6) | < 0.0001 |
|  | Copper (Cu) | 373 (52.7) | 335 (100.0) | 1460 (350) | 1505.0 (422.5) | 1410.0 (277.5) | NA | 1450.0 (345.0) | 1480.0 (357.5) | 0.076 |
|  | Mercury (Hg) | 191 (27.0) | 507 (98.1) | 2.6 (3.3) | 0.9 (0.8) | 3.0 (2.1) | 3.9 (4.1) | 0.7 (0.6) | 3.0 (2.6) | < 0.0001 |
|  | Manganese (Mn) | 373 (52.7) | 335 (100.0) | 11.7 (5.7) | 12.4 (6.1) | 10.4 (4.8) | NA | 13.7 (5.6) | 10.8 (5.3) | < 0.0001 |
|  | Molybdenum (Mo) | 373 (52.7) | 335 (100.0) | 0.6 (0.3) | 0.6 (0.2) | 0.6 (0.2) | NA | 0.5 (0.2) | 0.7 (0.3) | < 0.0001 |
|  | Lead (Pb) | 373 (52.7) | 335 (100.0) | 10.4 (6.5) | 7.7 (5.9) | 11.9 (6.9) | NA | 10.9 (6.9) | 10.1 (5.5) | 0.009 |
|  | Thallium (Tl) | 373 (52.7) | 5 (1.5) | < LOD | < LOD | < LOD | < LOD | < LOD | < LOD | NA |
| OCs | Dichlorodiphenyldichloroethylene (DDE) | 87 (12.3) | 620 (99.8) | 917.5 (1229) | 352.1 (958.4) | 932.7 (879.6) | 668.6 (560.1) | NA | 1921.5 (2129.1) | < 0.0001 |
|  | Dichlorodiphenyltrichloroethane (DDT) | 87 (12.3) | 298 (48.0) | 28.6 (50.8) | 15.7 (30.2) | 39.6 (50.5) | NA | NA | 13.7 (51.2) | < 0.0001 |
|  | Hexachlorobenzene (HCB) | 87 (12.3) | 611 (98.4) | 131.2 (137.1) | 61.1 (26.8) | 140.0 (71.0) | 237.3 (225.2) | NA | 78.2 (56.8) | < 0.0001 |
|  | Polychlorinated biphenyl-118 (PCB-118) | 87 (12.3) | 401 (64.6) | 36.5 (80.4) | 18.5 (11.6) | 103.8 (73.3) | 79.3 (4.0) | NA | 17.4 (11.4) | < 0.0001 |
|  | Polychlorinated biphenyl-138 (PCB-138) | 87 (12.3) | 583 (93.9) | 116.4 (183.6) | 40.1 (34.4) | 357.8 (252.9) | 109.0 (83.2) | NA | 68.4 (53.4) | < 0.0001 |
|  | Polychlorinated biphenyl-153 (PCB-153) | 87 (12.3) | 617 (99.4) | 217.5 (334.8) | 95.8 (72.4) | 700.8 (465.9) | 203.8 (141.0) | NA | 121.2 (99.8) | < 0.0001 |
|  | Polychlorinated biphenyl-170 (PCB-170) | 305 (43.1) | 400 (99.3) | 64.7 (159.6) | 13.1 (18.4) | 196.6 (168.3) | NA | NA | 35.4 (30.1) | < 0.0001 |
|  | Polychlorinated biphenyl-180 (PCB-180) | 87 (12.3) | 595 (95.8) | 145.7 (253.9) | 48.5 (55.2) | 520.5 (419.5) | 145.0 (100.0) | NA | 69.4 (55.1) | < 0.0001 |
| OP pesticide metabolites | Diethyl dithiophosphate (DEDTP) | 89 (12.6) | 13 (2.1) | < LOD | < LOD | < LOD | < LOD | NA | < LOD | NA |
|  | Diethyl phosphate (DEP) | 89 (12.6) | 606 (97.9) | 3.2 (5.6) | 2.2 (2.5) | 3.6 (5.9) | 3.2 (4.8) | NA | 2.8 (6.8) | 0.025 |
|  | Diethyl thiophosphate (DETP) | 89 (12.6) | 330 (53.3) | 0.3 (2.4) | 0.2 (1.8) | 0.9 (2.5) | 0.1 (1.9) | NA | 0.1 (2.6) | 0.122 |
|  | Dimethyl dithiophosphate (DMDTP) | 89 (12.6) | 357 (57.7) | 1.6 (6.2) | 0.2 (0.0) | 2.7 (7.0) | 0.2 (4.7) | NA | 3.4 (8.8) | < 0.0001 |
|  | Dimethyl phosphate (DMP) | 89 (12.6) | 583 (94.2) | 9.3 (13.7) | 3.5 (4.0) | 11.3 (16.6) | 9.9 (12.9) | NA | 8.9 (13.5) | < 0.0001 |
|  | Dimethyl thiophosphate (DMTP) | 89 (12.6) | 564 (91.1) | 5.2 (10.6) | 1.5 (5.0) | 6.4 (11.1) | 5.0 (10.3) | NA | 5.6 (13.8) | < 0.0001 |
| PBDEs | Polybrominated diphenyl ether-47 (PBDE-47) | 472 (66.7) | 152 (64.4) | 5.6 (8.6) | 8.9 (7.6) | 4.9 (7.7) | NA | NA | NA | < 0.0001 |
|  | Polybrominated diphenyl ether-153 (PBDE-153) | 304 (42.9) | 266 (65.8) | 6.7 (7.7) | 5.4 (6.9) | 5.9 (4.8) | NA | NA | 17.5 (25.7) | < 0.0001 |
| PFASs | Perfluorohexane sulfonate (PFHXS) | 24 ( 3.4) | 655 (95.8) | 0.6 (0.7) | 0.5 (0.6) | 1.0 (0.8) | 0.8 (0.5) | 0.4 (0.2) | 0.3 (0.2) | < 0.0001 |
|  | Perfluorononanoate (PFNA) | 24 ( 3.4) | 660 (96.5) | 0.9 (0.7) | 0.4 (0.3) | 0.9 (0.5) | 0.8 (0.5) | 0.7 (0.4) | 1.4 (0.8) | < 0.0001 |
|  | Perfluorooctanoate (PFOA) | 24 ( 3.4) | 680 (99.4) | 2.7 (2) | 2.1 (1.2) | 3.5 (1.7) | 2.8 (1.9) | 1.0 (0.9) | 2.4 (1.4) | < 0.0001 |
|  | Perfluorooctane sulfonate (PFOS) | 24 ( 3.4) | 684 (100.0) | 6.5 (5.5) | 3.8 (3.4) | 13.4 (9.8) | 6.2 (3.8) | 4.4 (2.4) | 5.3 (2.7) | < 0.0001 |
|  | Perfluoroundecanoate (PFUNDA) | 229 (32.3) | 469 (97.9) | 0.2 (0.2) | 0.0 (0.1) | 0.2 (0.1) | 0.3 (0.3) | 0.2 (0.1) | 0.3 (0.2) | < 0.0001 |
| Phenols | Bisphenol A (BPA) | 90 (12.7) | 614 (99.4) | 2.3 (3.1) | 1.6 (2.0) | 2.5 (2.7) | 2.4 (3.9) | NA | 2.5 (3.0) | 0.043 |
|  | N-butyl paraben (BUPA) | 90 (12.7) | 599 (96.9) | 1.7 (9.1) | 0.3 (3.6) | 1.3 (7.1) | 4.0 (9.1) | NA | 1.3 (10.2) | < 0.0001 |
|  | Ethyl paraben (ETPA) | 90 (12.7) | 599 (96.9) | 6.3 (23.7) | 1.4 (13.9) | 3.5 (12.3) | 12.7 (36.1) | NA | 5.4 (22.5) | < 0.0001 |
|  | Methyl paraben (MEPA) | 90 (12.7) | 618 (100.0) | 130.2 (362.2) | 98.5 (259.0) | 86.8 (254.6) | 213.6 (491.9) | NA | 143.2 (429.0) | < 0.0001 |
|  | Oxybenzone (OXBE) | 90 (12.7) | 611 (98.9) | 3 (14.4) | 19.7 (46.1) | 1.8 (4.8) | 3.2 (20.0) | NA | 4.4 (13.6) | < 0.0001 |
|  | Propyl paraben (PRPA) | 90 (12.7) | 605 (97.9) | 25.4 (92.2) | 34.8 (100.5) | 11.2 (39.0) | 46.6 (123.5) | NA | 25.1 (118.3) | < 0.0001 |
|  | Triclosan (TRCS) | 90 (12.7) | 604 (97.7) | 15.9 (99.2) | 2.3 (31.5) | 28.4 (153.6) | 33.4 (127.3) | NA | 2.0 (28.8) | < 0.0001 |
| Phthalate metabolites | Mono benzyl phthalate (MBzP) | 86 (12.1) | 620 (99.7) | 10.1 (18.1) | 3.4 (4.9) | 21.5 (34.6) | 8.1 (13.3) | NA | 5.6 (11.0) | < 0.0001 |
|  | Mono-2-ethyl 5-carboxypentyl phthalate (MECPP) | 256 (36.2) | 452 (100.0) | 38.7 (42.6) | 24.7 (30.9) | 43.7 (46.8) | 31.7 (27.3) | NA | 39.4 (42.8) | < 0.0001 |
|  | Mono-2-ethyl-5-hydroxyhexyl phthalate (MEHHP) | 86 (12.1) | 622 (100.0) | 22.6 (29.4) | 8.3 (12.2) | 29.7 (33.4) | 19.9 (19.4) | NA | 24.1 (33.2) | < 0.0001 |
|  | Mono-2-ethylhexyl phthalate (MEHP) | 86 (12.1) | 622 (100.0) | 7.7 (12.6) | 2.8 (4.5) | 9.3 (12.2) | 6.7 (10.2) | NA | 8.4 (16.6) | < 0.0001 |
|  | Mono-2-ethyl-5-oxohexyl phthalate (MEOHP) | 86 (12.1) | 622 (100.0) | 17 (20.5) | 8.8 (11.8) | 22.2 (25.7) | 14.9 (18.3) | NA | 15.6 (20.2) | < 0.0001 |
|  | Monoethyl phthalate (MEP) | 86 (12.1) | 621 (99.8) | 192.5 (393.9) | 170.5 (295.5) | 123.3 (234.4) | 331.5 (617.1) | NA | 145.7 (316.6) | < 0.0001 |
|  | Mono-iso-butyl phthalate (MiBP) | 86 (12.1) | 622 (100.0) | 42.1 (58.4) | 33.1 (36.8) | 56.7 (59.1) | 28.1 (35.0) | NA | 52.6 (74.0) | < 0.0001 |
|  | Mono-n-butyl phthalate (MnBP) | 86 (12.1) | 622 (100.0) | 30.2 (43.3) | 22.0 (25.5) | 46.4 (50.1) | 23.8 (34.8) | NA | 21.8 (48.8) | < 0.0001 |
|  | Mono-4-methyl-7-hydroxyoctyl phthalate (ohMiNP) | 256 (36.2) | 403 (89.2) | 0.8 (0.7) | 0.5 (0.5) | 0.7 (0.5) | 1.2 (0.9) | NA | 0.8 (1.0) | < 0.0001 |
|  | Mono-4-methyl-7-oxooctyl phthalate (oxo-MiNP) | 256 (36.2) | 421 (93.1) | 0.8 (1) | 0.9 (1.3) | 0.8 (0.7) | 1.0 (1.2) | NA | 0.9 (1.5) | 0.076 |
|  | Cotinine | 85 (12.0) | 339 (54.4) | 8.8 (36.4) | 2.8 (15.7) | 2.5 (26.9) | 6.0 (23.9) | NA | 24.4 (90.7) | < 0.0001 |

Distributions are reported for raw (i.e. non-imputed and non-transformed) exposure values.

^a^ Kruskal-Wallis test was applied on continuous variables. Abbreviations: BiB = Born in Bradford. EDEN = Étude des Déterminants Pré et Postnatals du Développement et de la Santé de l’Enfant. INMA = Infancia y Medio Ambiente. KANC = Kaunas Cohort. RHEA = Mother-Child Cohort in Crete. LOD = limit of detection. IQR = inter-quartile range. NA = not applicable. OCs = organochlorine compounds. OP = organophosphate. PBDEs = polybrominated diphenyl ethers. PFASs = per- and polyfluoroalkyl substances.

**Appendix Table 6:** Adjusted associations between prenatal exposure to environmental contaminants and SDQ *externalising* score (n = 708).

| **Exposure family** | **Exposure** | **ExWAS** | | | **ExWAS with cohort-exposure interaction^a^** | | | | |  |
| --- | --- | --- | --- | --- | --- | --- | --- | --- | --- | --- |
|  |  | **IRR (95%CI)^b^** | **p value** | **FWER**  **p value** | **IRR (95%CI)^b^** | | | | | **I^2^** |
|  |  |  |  |  | **BiB** | **EDEN** | **INMA** | **KANC** | **RHEA** |  |
| Metals and semi-metals (essential and toxic elements) | Inorganic arsenic (As) | 0.98 (0.86; 1.12) | 0.781 | 1 | 0.84 (0.45;1.57) | 0.96 (0.76;1.21) | 1.00 (0.79;1.25) | 0.93 (0.59;1.46) | 1.01 (0.85;1.21) | <0.001 |
|  | Cadmium (Cd) | 1.08 (0.97; 1.20) | 0.182 | 1 | 1.17 (0.76;1.80) | 1.11 (0.92;1.33) | 1.03 (0.88;1.22) | 1.09 (0.92;1.29) | 1.12 (0.97;1.29) | <0.001 |
|  | Cobalt (Co) | 1.10 (0.98; 1.24) | 0.112 | 1 | 1.21 (0.85;1.73) | 1.21 (0.99;1.47) | 1.04 (0.86;1.26) | 0.99 (0.75;1.30) | 1.09 (0.92;1.30) | <0.001 |
|  | Caesium (Cs) | 0.96 (0.86; 1.08) | 0.480 | 1 | 0.90 (0.55;1.48) | 0.94 (0.78;1.13) | 0.95 (0.80;1.12) | 1.06 (0.83;1.36) | 1.01 (0.83;1.23) | <0.001 |
|  | Copper (Cu) | 0.90 (0.82; 0.98) | 0.021 | 0.643 | 0.63 (0.41;0.97) | 0.87 (0.74;1.03) | 0.92 (0.79;1.07) | 0.95 (0.80;1.12) | 0.92 (0.81;1.04) | <0.001 |
|  | Mercury (Hg) | 0.96 (0.86; 1.07) | 0.413 | 1 | 0.94 (0.50;1.77) | 0.99 (0.81;1.20) | 0.97 (0.82;1.15) | 0.76 (0.58;0.99) | 0.98 (0.82;1.17) | <0.001 |
|  | Manganese (Mn) | 1.00 (0.90; 1.12) | 0.955 | 1 | 0.91 (0.61;1.36) | 1.03 (0.84;1.26) | 0.99 (0.81;1.21) | 1.03 (0.81;1.31) | 0.99 (0.85;1.15) | <0.001 |
|  | Molybdenum (Mo) | 1.00 (0.95; 1.04) | 0.840 | 1 | 0.94 (0.74;1.20) | 1.01 (0.94;1.07) | 0.99 (0.92;1.07) | 0.98 (0.86;1.11) | 0.95 (0.85;1.06) | <0.001 |
|  | Lead (Pb) | 1.03 (0.95; 1.11) | 0.524 | 1 | 0.79 (0.56;1.10) | 1.03 (0.90;1.18) | 1.04 (0.94;1.15) | 1.14 (0.95;1.38) | 0.98 (0.87;1.09) | <0.001 |
| OCs | Dichlorodiphenyldichloroethylene (DDE) | 0.93 (0.86; 1.01) | 0.095 | 1 | 0.64 (0.48;0.85) | 0.92 (0.79;1.08) | 0.98 (0.84;1.13) | 0.98 (0.73;1.31) | 0.98 (0.85;1.12) | 0.393 |
|  | Dichlorodiphenyltrichloroethane (DDT) | 0.92 (0.84; 1.00) | 0.045 | 1 | 0.73 (0.59;0.91) | 0.83 (0.71;0.95) | 0.98 (0.82;1.18) | 0.96 (0.75;1.24) | 0.97 (0.84;1.11) | 0.417 |
|  | Hexachlorobenzene (HCB) | 0.93 (0.86; 1.01) | 0.073 | 1 | 0.50 (0.25;1.01) | 0.90 (0.80;1.02) | 0.94 (0.82;1.07) | 0.96 (0.73;1.27) | 0.99 (0.83;1.19) | <0.001 |
|  | Polychlorinated biphenyl-118 (PCB-118) | 0.89 (0.76; 1.04) | 0.153 | 1 | 0.31 (0.09;1.02) | 0.72 (0.53;0.96) | 0.96 (0.77;1.20) | 0.91 (0.59;1.40) | 0.87 (0.66;1.15) | 0.032 |
|  | Polychlorinated biphenyl-138 (PCB-138) | 0.88 (0.79; 0.99) | 0.035 | 1 | 0.60 (0.37;0.96) | 0.91 (0.76;1.09) | 0.84 (0.69;1.02) | 0.94 (0.68;1.30) | 0.91 (0.74;1.11) | <0.001 |
|  | Polychlorinated biphenyl-153 (PCB-153) | 0.88 (0.77; 1.01) | 0.065 | 1 | 0.57 (0.33;0.98) | 0.95 (0.77;1.18) | 0.80 (0.66;0.98) | 0.94 (0.64;1.37) | 0.93 (0.74;1.17) | <0.001 |
|  | Polychlorinated biphenyl-170 (PCB-170) | 0.93 (0.83; 1.04) | 0.209 | 1 | 0.58 (0.36;0.95) | 0.98 (0.82;1.17) | 0.89 (0.74;1.07) | 0.99 (0.76;1.28) | 0.96 (0.76;1.21) | <0.001 |
|  | Polychlorinated biphenyl-180 (PCB-180) | 0.92 (0.81; 1.06) | 0.253 | 1 | 0.62 (0.42;0.92) | 1.02 (0.84;1.24) | 0.82 (0.65;1.04) | 0.99 (0.72;1.35) | 0.94 (0.73;1.20) | 0.201 |
| OP pesticide metabolites | Diethyl phosphate (DEP) | 1.03 (0.97; 1.09) | 0.337 | 1 | 1.07 (0.74;1.54) | 1.05 (0.94;1.19) | 0.99 (0.90;1.08) | 1.02 (0.83;1.27) | 1.08 (0.96;1.22) | <0.001 |
|  | Diethyl thiophosphate (DETP) | 0.99 (0.91; 1.08) | 0.784 | 1 | 0.73 (0.47;1.13) | 1.07 (0.91;1.25) | 0.85 (0.73;0.99) | 1.02 (0.76;1.36) | 1.09 (0.94;1.26) | 0.487 |
|  | Dimethyl dithiophosphate (DMDTP) | 1.03 (0.94; 1.13) | 0.545 | 1 | 0.84 (0.45;1.54) | 1.06 (0.90;1.24) | 1.02 (0.88;1.19) | 1.00 (0.74;1.34) | 1.04 (0.87;1.25) | <0.001 |
|  | Dimethyl phosphate (DMP) | 1.03 (0.98; 1.09) | 0.243 | 1 | 0.72 (0.55;0.95) | 1.11 (1.01;1.22) | 1.01 (0.93;1.10) | 1.04 (0.84;1.29) | 1.03 (0.93;1.14) | 0.319 |
|  | Dimethyl thiophosphate (DMTP) | 1.03 (0.98; 1.08) | 0.274 | 1 | 0.96 (0.78;1.17) | 1.09 (0.99;1.20) | 0.98 (0.90;1.05) | 1.04 (0.84;1.29) | 1.10 (0.97;1.24) | 0.299 |
| PBDEs | Polybrominated diphenyl ether-153 (PBDE-153) | 1.00 (0.89; 1.12) | 0.983 | 1 | 1.02 (0.63;1.65) | 0.95 (0.79;1.14) | 1.03 (0.83;1.26) | 0.97 (0.71;1.34) | 1.02 (0.80;1.29) | <0.001 |
|  | Polybrominated diphenyl ether-47 (PBDE-47) | 0.95 (0.88; 1.02) | 0.154 | 1 | 0.92 (0.67;1.26) | 0.85 (0.75;0.97) | 0.96 (0.84;1.10) | 0.98 (0.81;1.18) | 0.98 (0.87;1.10) | 0.022 |
| PFASs | Perfluorohexane sulfonate (PFHxS) | 0.96 (0.88; 1.05) | 0.377 | 1 | 1.05 (0.76;1.45) | 0.86 (0.73;1.01) | 0.97 (0.86;1.09) | 0.96 (0.71;1.29) | 1.10 (0.86;1.41) | <0.001 |
|  | Perfluorononanoate (PFNA) | 0.97 (0.92; 1.02) | 0.239 | 1 | 0.96 (0.78;1.18) | 0.86 (0.75;1.00) | 0.98 (0.92;1.04) | 0.97 (0.81;1.17) | 1.09 (0.91;1.31) | <0.001 |
|  | Perfluorooctanoate (PFOA) | 1.00 (0.94; 1.06) | 0.883 | 1 | 1.28 (0.89;1.84) | 0.87 (0.73;1.04) | 0.97 (0.91;1.05) | 1.06 (0.88;1.27) | 1.08 (0.91;1.29) | 0.067 |
|  | Perfluorooctane sulfonate (PFOS) | 0.97 (0.91; 1.03) | 0.299 | 1 | 1.14 (0.85;1.52) | 0.90 (0.80;1.02) | 0.96 (0.89;1.04) | 0.96 (0.78;1.18) | 1.07 (0.87;1.31) | <0.001 |
|  | Perfluoroundecanoate (PFUnDA) | 0.92 (0.84; 0.99) | 0.034 | 1 | 1.04 (0.84;1.30) | 0.83 (0.72;0.96) | 0.91 (0.80;1.04) | 0.97 (0.75;1.25) | 0.95 (0.78;1.16) | <0.001 |
| Phenols | Bisphenol A (BPA) | 1.06 (1.01; 1.12) | 0.028 | 0.849 | 1.05 (0.76;1.44) | 1.19 (1.05;1.35) | 1.02 (0.94;1.10) | 1.01 (0.85;1.19) | 1.10 (1.00;1.22) | 0.348 |
|  | N-butyl paraben (BUPA) | 0.97 (0.89; 1.06) | 0.514 | 1 | 0.62 (0.43;0.89) | 0.96 (0.81;1.13) | 1.00 (0.87;1.16) | 0.98 (0.71;1.34) | 1.03 (0.89;1.20) | <0.001 |
|  | Ethyl paraben (ETPA) | 0.96 (0.89; 1.05) | 0.380 | 1 | 0.92 (0.66;1.29) | 0.99 (0.85;1.16) | 0.93 (0.81;1.07) | 0.96 (0.73;1.26) | 0.99 (0.85;1.15) | <0.001 |
|  | Methyl paraben (MEPA) | 0.97 (0.91; 1.04) | 0.406 | 1 | 0.96 (0.69;1.35) | 0.96 (0.84;1.09) | 0.97 (0.85;1.10) | 1.01 (0.81;1.26) | 0.97 (0.87;1.08) | <0.001 |
|  | Oxybenzone (OXBE) | 1.03 (0.97; 1.10) | 0.310 | 1 | 0.89 (0.65;1.22) | 1.07 (0.93;1.22) | 1.06 (0.95;1.17) | 0.99 (0.81;1.21) | 1.04 (0.91;1.17) | <0.001 |
|  | Propyl paraben (PRPA) | 0.96 (0.90; 1.02) | 0.173 | 1 | 0.73 (0.56;0.95) | 0.95 (0.84;1.08) | 0.94 (0.83;1.08) | 1.00 (0.81;1.24) | 0.98 (0.89;1.09) | <0.001 |
|  | Triclosan (TRCS) | 1.03 (0.93; 1.12) | 0.593 | 1 | 1.59 (1.01;2.50) | 0.93 (0.79;1.10) | 1.02 (0.86;1.20) | 1.00 (0.73;1.36) | 1.09 (0.92;1.28) | <0.001 |
| Phthalate metabolites | Mono benzyl phthalate (MbzP) | 1.00 (0.93; 1.08) | 0.938 | 1 | 1.03 (0.66;1.60) | 0.97 (0.85;1.12) | 1.01 (0.89;1.15) | 1.01 (0.84;1.21) | 1.01 (0.88;1.15) | <0.001 |
|  | Mono-2-ethyl 5-carboxypentyl phthalate (MECPP) | 1.00 (0.93; 1.08) | 0.898 | 1 | 0.50 (0.31;0.82) | 1.01 (0.90;1.14) | 1.02 (0.88;1.18) | 0.99 (0.80;1.23) | 1.04 (0.92;1.19) | <0.001 |
|  | Mono-2-ethyl-5-hydroxyhexyl phthalate (MEHHP) | 1.00 (0.94; 1.07) | 0.974 | 1 | 0.46 (0.29;0.71) | 1.01 (0.90;1.15) | 1.03 (0.92;1.14) | 1.00 (0.82;1.21) | 1.02 (0.91;1.15) | <0.001 |
|  | Mono-2-ethylhexyl phthalate (MEHP) | 0.99 (0.93; 1.06) | 0.844 | 1 | 0.42 (0.28;0.63) | 1.03 (0.91;1.16) | 1.02 (0.91;1.14) | 1.00 (0.80;1.26) | 1.01 (0.90;1.14) | 0.939 |
|  | Mono-2-ethyl-5-oxohexyl phthalate (MEOHP) | 0.99 (0.93; 1.06) | 0.778 | 1 | 0.41 (0.26;0.65) | 0.98 (0.87;1.11) | 1.03 (0.93;1.15) | 0.99 (0.81;1.20) | 1.02 (0.91;1.14) | 0.931 |
|  | Monoethyl phthalate (MEP) | 1.02 (0.95; 1.11) | 0.552 | 1 | 0.66 (0.44;0.99) | 0.95 (0.83;1.09) | 1.14 (0.99;1.30) | 1.00 (0.77;1.29) | 1.06 (0.92;1.23) | 0.385 |
|  | Mono-iso-butyl phthalate (MiBP) | 1.00 (0.94; 1.07) | 0.996 | 1 | 0.72 (0.51;1.02) | 1.01 (0.90;1.15) | 1.01 (0.91;1.12) | 0.98 (0.80;1.20) | 1.02 (0.91;1.15) | <0.001 |
|  | Mono-n-butyl phthalate (MnBP) | 1.06 (1.00; 1.13) | 0.048 | 1 | 0.88 (0.56;1.40) | 1.15 (1.03;1.28) | 1.04 (0.94;1.15) | 1.02 (0.85;1.22) | 1.05 (0.94;1.18) | <0.001 |
|  | Mono-4-methyl-7-hydroxyoctyl phthalate (ohMiNP) | 1.01 (0.97; 1.06) | 0.642 | 1 | 0.80 (0.63;1.02) | 0.95 (0.86;1.04) | 1.01 (0.93;1.11) | 1.01 (0.87;1.17) | 1.06 (0.99;1.13) | 0.341 |
|  | Mono-4-methyl-7-oxooctyl phthalate (oxo-MiNP) | 1.02 (0.96; 1.07) | 0.597 | 1 | 1.07 (0.78;1.46) | 0.99 (0.89;1.09) | 1.03 (0.91;1.16) | 1.02 (0.84;1.23) | 1.02 (0.94;1.10) | <0.001 |
|  | Cotinine | 1.02 (0.95; 1.10) | 0.529 | 1 | 1.09 (0.81;1.48) | 0.96 (0.86;1.08) | 1.01 (0.90;1.12) | 1.03 (0.84;1.27) | 1.13 (0.98;1.30) | 0.027 |

. ^a^ For each exposure we fitted a negative binomial regression model with adjustment factors and a cohort-exposure interaction term, from which the I² statistic measuring the between-cohort heterogeneity of exposure association with SDQ score was extracted. I^2^ interpretation: I^2^ < 0.3 = low heterogeneity, 0.3 ≤ I^2^ < 0.6 = moderate heterogeneity, I^2^ ≥ 0.6 = substantial to high heterogeneity. Missing values were multiply imputed before model fitting. ^b^ IRRs are reported with 95%CIs and correspond to the change in the probability of the SDQ scores increasing by one unit for an IQR change in the log_2_ or ln (cotinine only) of the biomarker concentration in maternal blood or urine. Models were adjusted for cohort, season of conception, child sex and age at SDQ assessment, parity, and maternal factors: education level, work status, age, pre- pregnancy BMI, and prenatal active smoking status. Abbreviations: BiB = Born in Bradford. EDEN = Étude des Déterminants Pré et Postnatals du Développement et de la Santé de l’Enfant. INMA = Infancia y Medio Ambiente. KANC = Kaunas Cohort. RHEA = Mother-Child Cohort in Crete. BMI = body mass index. CI = confidence interval of the IRR estimate. ExWAS = exposome-wide association study. FWER = family wise error rate adjustment. IQR = inter-quartile range. IRR = incidence rate ratio. SDQ = Strengths and Difficulties Questionnaire. OCs = organochlorine compounds. OP = organophosphate. PBDEs = polybrominated diphenyl ethers. PFASs = per- and polyfluoroalkyl substances.

**Appendix Table 7:** Adjusted associations between prenatal exposure to environmental contaminants and SDQ *internalising* score (n = 708).

| **Exposure family** |  | **ExWAS** | | | **ExWAS with cohort-exposure interaction^a^** | | | | |  |
| --- | --- | --- | --- | --- | --- | --- | --- | --- | --- | --- |
|  | **Exposure** |  |  | **FWER** | **IRR (95%CI)^b^** | | | | | **I^2^** |
|  |  | **IRR (95%CI)^b^** | **p value** | **p value** | **BiB** | **EDEN** | **INMA** | **KANC** | **RHEA** |  |
| Metals and semi-metals (essential and toxic elements) | Inorganic arsenic (As) | 0.97 (0.83; 1.14) | 0.692 | 1 | 0.93 (0.50;1.74) | 1.03 (0.78;1.35) | 0.98 (0.73;1.33) | 0.96 (0.54;1.71) | 0.89 (0.70;1.13) | <0.001 |
|  | Cadmium (Cd) | 1.04 (0.92; 1.17) | 0.547 | 1 | 0.92 (0.56;1.50) | 0.98 (0.81;1.19) | 1.07 (0.88;1.30) | 1.02 (0.83;1.26) | 1.13 (0.94;1.36) | <0.001 |
|  | Cobalt (Co) | 1.10 (0.96; 1.27) | 0.179 | 1 | 1.50 (1.02;2.21) | 1.06 (0.85;1.33) | 1.08 (0.84;1.38) | 0.77 (0.54;1.09) | 1.23 (0.98;1.55) | 0.420 |
|  | Caesium (Cs) | 1.00 (0.87; 1.15) | 0.986 | 1 | 0.68 (0.41;1.14) | 0.99 (0.81;1.21) | 1.03 (0.82;1.29) | 1.24 (0.91;1.69) | 0.94 (0.72;1.22) | <0.001 |
|  | Copper (Cu) | 0.96 (0.86; 1.07) | 0.468 | 1 | 1.09 (0.72;1.66) | 0.91 (0.74;1.10) | 0.97 (0.80;1.18) | 0.88 (0.71;1.09) | 1.06 (0.89;1.26) | <0.001 |
|  | Mercury (Hg) | 0.97 (0.85; 1.11) | 0.642 | 1 | 0.64 (0.33;1.26) | 1.00 (0.80;1.24) | 0.96 (0.77;1.20) | 1.14 (0.80;1.62) | 0.94 (0.74;1.19) | <0.001 |
|  | Manganese (Mn) | 1.11 (0.98; 1.26) | 0.099 | 1 | 1.07 (0.70;1.63) | 1.07 (0.87;1.32) | 1.12 (0.87;1.44) | 1.03 (0.76;1.40) | 1.19 (0.98;1.44) | <0.001 |
|  | Molybdenum (Mo) | 0.97 (0.92; 1.03) | 0.386 | 1 | 0.86 (0.66;1.12) | 0.98 (0.90;1.07) | 0.99 (0.89;1.09) | 0.91 (0.77;1.07) | 0.95 (0.82;1.10) | <0.001 |
|  | Lead (Pb) | 0.95 (0.87; 1.05) | 0.309 | 1 | 0.89 (0.61;1.30) | 0.93 (0.79;1.08) | 0.96 (0.84;1.09) | 1.10 (0.86;1.41) | 0.94 (0.81;1.09) | <0.001 |
| OCs | Dichlorodiphenyldichloroethylene (DDE) | 0.99 (0.89; 1.10) | 0.836 | 1 | 1.25 (0.97;1.62) | 0.87 (0.71;1.06) | 0.93 (0.77;1.13) | 0.98 (0.68;1.42) | 1.03 (0.86;1.25) | 0.278 |
|  | Dichlorodiphenyltrichloroethane (DDT) | 1.01 (0.91; 1.12) | 0.899 | 1 | 1.15 (0.90;1.47) | 0.96 (0.80;1.16) | 1.01 (0.81;1.27) | 1.01 (0.74;1.38) | 0.93 (0.77;1.11) | <0.001 |
|  | Hexachlorobenzene (HCB) | 0.97 (0.87; 1.07) | 0.531 | 1 | 1.49 (0.74;2.97) | 0.91 (0.79;1.06) | 0.98 (0.83;1.16) | 0.95 (0.66;1.37) | 1.06 (0.84;1.34) | <0.001 |
|  | Polychlorinated biphenyl-118 (PCB-118) | 0.95 (0.77; 1.17) | 0.595 | 1 | 1.42 (0.46;4.39) | 0.96 (0.66;1.40) | 0.91 (0.67;1.23) | 0.93 (0.54;1.61) | 1.09 (0.75;1.58) | <0.001 |
|  | Polychlorinated biphenyl-138 (PCB-138) | 0.96 (0.83; 1.11) | 0.577 | 1 | 0.96 (0.60;1.54) | 0.95 (0.75;1.20) | 0.89 (0.69;1.14) | 1.00 (0.64;1.56) | 1.03 (0.79;1.35) | <0.001 |
|  | Polychlorinated biphenyl-153 (PCB-153) | 0.96 (0.81; 1.14) | 0.657 | 1 | 1.36 (0.79;2.33) | 0.96 (0.73;1.25) | 0.88 (0.68;1.13) | 0.96 (0.58;1.61) | 1.02 (0.76;1.38) | <0.001 |
|  | Polychlorinated biphenyl-170 (PCB-170) | 0.97 (0.84; 1.12) | 0.663 | 1 | 1.13 (0.72;1.78) | 0.97 (0.78;1.21) | 0.96 (0.77;1.20) | 0.98 (0.70;1.38) | 0.90 (0.66;1.23) | <0.001 |
|  | Polychlorinated biphenyl-180 (PCB-180) | 0.95 (0.80; 1.13) | 0.539 | 1 | 1.18 (0.80;1.73) | 0.98 (0.76;1.26) | 0.82 (0.61;1.11) | 0.97 (0.64;1.47) | 0.86 (0.63;1.19) | <0.001 |
| OP pesticide metabolites | Diethyl phosphate (DEP) | 1.01 (0.93; 1.09) | 0.842 | 1 | 1.42 (0.98;2.05) | 1.05 (0.90;1.22) | 0.95 (0.84;1.06) | 1.05 (0.79;1.39) | 1.01 (0.86;1.18) | <0.001 |
|  | Diethyl thiophosphate (DETP) | 1.11 (1.00; 1.24) | 0.053 | 1 | 2.04 (1.33;3.12) | 1.12 (0.91;1.36) | 0.98 (0.81;1.18) | 1.15 (0.78;1.67) | 1.11 (0.92;1.33) | 0.612 |
|  | Dimethyl dithiophosphate (DMDTP) | 0.97 (0.86; 1.09) | 0.577 | 1 | 1.28 (0.75;2.20) | 1.05 (0.86;1.30) | 0.86 (0.70;1.04) | 1.00 (0.70;1.43) | 0.97 (0.76;1.22) | <0.001 |
|  | Dimethyl phosphate (DMP) | 1.01 (0.95; 1.08) | 0.753 | 1 | 1.40 (1.02;1.93) | 0.98 (0.87;1.10) | 1.01 (0.91;1.13) | 1.04 (0.79;1.38) | 0.98 (0.86;1.12) | <0.001 |
|  | Dimethyl thiophosphate (DMTP) | 1.04 (0.97; 1.11) | 0.259 | 1 | 1.50 (1.20;1.86) | 1.08 (0.95;1.22) | 0.98 (0.89;1.08) | 0.99 (0.75;1.29) | 0.97 (0.83;1.13) | 0.766 |
| PBDEs | Polybrominated diphenyl ether-153 (PBDE-153) | 1.05 (0.92; 1.21) | 0.473 | 1 | 0.93 (0.58;1.49) | 1.18 (0.94;1.50) | 1.03 (0.80;1.34) | 0.99 (0.65;1.51) | 1.04 (0.76;1.43) | <0.001 |
|  | Polybrominated diphenyl ether-47 (PBDE-47) | 0.99 (0.90; 1.08) | 0.800 | 1 | 0.92 (0.68;1.23) | 0.95 (0.80;1.12) | 1.02 (0.86;1.21) | 1.01 (0.79;1.29) | 0.98 (0.84;1.15) | <0.001 |
| PFASs | Perfluorohexane sulfonate (PFHxS) | 0.93 (0.84; 1.03) | 0.176 | 1 | 0.66 (0.46;0.93) | 0.98 (0.80;1.21) | 0.99 (0.85;1.15) | 0.91 (0.62;1.34) | 0.91 (0.67;1.24) | <0.001 |
|  | Perfluorononanoate (PFNA) | 0.96 (0.90; 1.02) | 0.147 | 1 | 0.78 (0.64;0.94) | 1.08 (0.90;1.29) | 0.97 (0.90;1.05) | 0.83 (0.65;1.05) | 1.06 (0.85;1.32) | 0.573 |
|  | Perfluorooctanoate (PFOA) | 0.97 (0.90; 1.04) | 0.406 | 1 | 0.67 (0.47;0.98) | 0.97 (0.78;1.20) | 0.98 (0.89;1.07) | 0.94 (0.74;1.19) | 1.06 (0.86;1.31) | <0.001 |
|  | Perfluorooctane sulfonate (PFOS) | 0.92 (0.85; 1.00) | 0.037 | 1 | 0.68 (0.51;0.91) | 1.02 (0.87;1.19) | 0.94 (0.85;1.05) | 0.75 (0.58;0.98) | 0.98 (0.76;1.27) | 0.569 |
|  | Perfluoroundecanoate (PFUnDA) | 0.93 (0.84; 1.03) | 0.161 | 1 | 1.06 (0.85;1.31) | 1.00 (0.83;1.21) | 0.87 (0.73;1.03) | 0.68 (0.49;0.95) | 1.04 (0.79;1.37) | 0.274 |
| Phenols | Bisphenol A (BPA) | 1.04 (0.98; 1.12) | 0.209 | 1 | 1.14 (0.83;1.55) | 1.16 (0.98;1.36) | 1.02 (0.93;1.13) | 0.98 (0.78;1.23) | 1.04 (0.92;1.19) | <0.001 |
|  | N-butyl paraben (BUPA) | 1.09 (0.98; 1.21) | 0.121 | 1 | 1.24 (0.89;1.72) | 1.03 (0.83;1.27) | 1.24 (1.02;1.51) | 0.99 (0.67;1.46) | 1.00 (0.82;1.21) | 0.111 |
|  | Ethyl paraben (ETPA) | 1.04 (0.94; 1.15) | 0.475 | 1 | 1.21 (0.88;1.68) | 1.02 (0.83;1.25) | 1.10 (0.91;1.31) | 0.95 (0.67;1.34) | 1.00 (0.82;1.21) | <0.001 |
|  | Methyl paraben (MEPA) | 0.98 (0.90; 1.07) | 0.697 | 1 | 0.95 (0.68;1.33) | 0.95 (0.80;1.12) | 1.04 (0.88;1.22) | 1.00 (0.76;1.31) | 0.97 (0.84;1.11) | <0.001 |
|  | Oxybenzone (OXBE) | 1.00 (0.92; 1.09) | 0.924 | 1 | 0.78 (0.57;1.06) | 0.94 (0.79;1.13) | 1.10 (0.96;1.26) | 0.98 (0.77;1.26) | 1.02 (0.87;1.20) | 0.134 |
|  | Propyl paraben (PRPA) | 0.98 (0.91; 1.07) | 0.675 | 1 | 0.92 (0.70;1.21) | 0.93 (0.80;1.09) | 1.02 (0.86;1.21) | 1.01 (0.77;1.30) | 1.00 (0.88;1.14) | <0.001 |
|  | Triclosan (TRCS) | 1.08 (0.96; 1.21) | 0.204 | 1 | 1.83 (1.15;2.90) | 1.12 (0.91;1.39) | 1.00 (0.81;1.25) | 1.04 (0.69;1.55) | 1.02 (0.82;1.25) | <0.001 |
| Phthalate metabolites | Mono benzyl phthalate (MbzP) | 1.04 (0.95; 1.14) | 0.393 | 1 | 0.75 (0.49;1.15) | 1.12 (0.94;1.33) | 1.15 (0.98;1.35) | 1.00 (0.78;1.27) | 0.94 (0.79;1.12) | 0.240 |
|  | Mono-2-ethyl 5-carboxypentyl phthalate (MECPP) | 1.02 (0.93; 1.13) | 0.616 | 1 | 0.84 (0.54;1.31) | 1.15 (0.99;1.34) | 0.98 (0.82;1.18) | 1.00 (0.75;1.31) | 0.99 (0.84;1.17) | 0.099 |
|  | Mono-2-ethyl-5-hydroxyhexyl phthalate (MEHHP) | 1.00 (0.92; 1.08) | 0.942 | 1 | 0.70 (0.47;1.04) | 1.11 (0.95;1.29) | 1.00 (0.87;1.16) | 0.98 (0.78;1.23) | 0.95 (0.82;1.10) | <0.001 |
|  | Mono-2-ethylhexyl phthalate (MEHP) | 1.03 (0.94; 1.12) | 0.563 | 1 | 0.81 (0.56;1.18) | 1.12 (0.96;1.31) | 1.00 (0.86;1.16) | 1.01 (0.75;1.36) | 1.01 (0.86;1.18) | <0.001 |
|  | Mono-2-ethyl-5-oxohexyl phthalate (MEOHP) | 1.01 (0.93; 1.09) | 0.863 | 1 | 0.76 (0.52;1.13) | 1.11 (0.95;1.29) | 1.02 (0.89;1.17) | 1.00 (0.78;1.26) | 0.95 (0.82;1.10) | <0.001 |
|  | Monoethyl phthalate (MEP) | 0.99 (0.90; 1.09) | 0.876 | 1 | 0.57 (0.38;0.86) | 1.00 (0.84;1.19) | 1.14 (0.96;1.36) | 0.94 (0.68;1.31) | 0.95 (0.79;1.15) | 0.595 |
|  | Mono-iso-butyl phthalate (MiBP) | 0.99 (0.92; 1.08) | 0.891 | 1 | 1.30 (0.96;1.78) | 0.92 (0.79;1.08) | 0.96 (0.84;1.10) | 0.99 (0.78;1.26) | 1.04 (0.90;1.21) | <0.001 |
|  | Mono-n-butyl phthalate (MnBP) | 1.03 (0.95; 1.11) | 0.488 | 1 | 0.86 (0.54;1.39) | 1.10 (0.95;1.26) | 0.98 (0.86;1.12) | 1.01 (0.82;1.26) | 1.03 (0.89;1.20) | <0.001 |
|  | Mono-4-methyl-7-hydroxyoctyl phthalate (ohMiNP) | 0.98 (0.93; 1.04) | 0.469 | 1 | 0.78 (0.61;1.00) | 0.97 (0.86;1.10) | 0.97 (0.87;1.09) | 1.00 (0.84;1.19) | 1.01 (0.93;1.10) | 0.001 |
|  | Mono-4-methyl-7-oxooctyl phthalate (oxo-MiNP) | 1.00 (0.93; 1.07) | 0.923 | 1 | 0.85 (0.60;1.20) | 1.04 (0.91;1.19) | 0.95 (0.80;1.12) | 0.99 (0.78;1.26) | 1.02 (0.92;1.13) | <0.001 |
|  | Cotinine | 0.97 (0.88; 1.06) | 0.519 | 1 | 0.76 (0.54;1.07) | 0.89 (0.77;1.02) | 0.98 (0.86;1.13) | 0.95 (0.72;1.25) | 1.14 (0.96;1.36) | 0.392 |

^a^ For each exposure we fitted a negative binomial regression model with adjustment factors and a cohort-exposure interaction term, from which the I² statistic measuring the between-cohort heterogeneity of exposure association with SDQ score was extracted. I^2^ interpretation: I^2^ < 0.3 = low heterogeneity, 0.3 ≤ I^2^ < 0.6 = moderate heterogeneity, I^2^ ≥ 0.6 = substantial to high heterogeneity. Missing values were multiply imputed before model fitting. ^b^ IRRs are reported with 95%CIs and correspond to the change in the probability of the SDQ scores increasing by one unit for an IQR change in the log_2_ or ln (cotinine only) of the biomarker concentration in maternal blood or urine. Regression models were adjusted for cohort, season of conception, child sex and age at SDQ assessment, parity, and maternal factors: education level, work status, age, pre- pregnancy BMI, and prenatal active smoking status. Abbreviations: BiB = Born in Bradford. EDEN = Étude des Déterminants Pré et Postnatals du Développement et de la Santé de l’Enfant. INMA = Infancia y Medio Ambiente. KANC = Kaunas Cohort. RHEA = Mother-Child Cohort in Crete. BMI = body mass index. CI = confidence interval of the IRR estimate. ExWAS = exposome-wide association study. FWER = family wise error rate adjustment. IQR = inter-quartile range. IRR = incidence rate ratio. SDQ = Strengths and Difficulties Questionnaire. OCs = organochlorine compounds. OP = organophosphate. PBDEs = polybrominated diphenyl ethers. PFASs = per- and polyfluoroalkyl substances.

#### References

Aurrekoetxea JJ, Murcia M, Rebagliato M, López MJ, Castilla AM, Santa-Marina L, et al. 2013. Determinants of self-reported smoking and misclassification during pregnancy, and analysis of optimal cut-off points for urinary cotinine: a cross-sectional study. BMJ Open 3:e002034; doi:10.1136/bmjopen-2012-002034. PMID: 23355667.

Caspersen IH, Kvalem HE, Haugen M, Brantsæter AL, Meltzer HM, Alexander J, et al. 2016. Determinants of plasma PCB, brominated flame retardants, and organochlorine pesticides in pregnant women and 3 year old children in The Norwegian Mother and Child Cohort Study. Environ Res 146:136–144; doi:10.1016/j.envres.2015.12.020. PMID: 26749444.

Cequier E, Sakhi AK, Haug LS, Thomsen C. 2016. Development of an ion-pair liquid chromatography-high resolution mass spectrometry method for determination of organophosphate pesticide metabolites in large-scale biomonitoring studies. J Chromatogr A 1454:32–41; doi:10.1016/j.chroma.2016.05.067. PMID: 27264744.

Goñi F, López R, Etxeandia A, Millán E, Amiano P. 2007. High throughput method for the determination of organochlorine pesticides and polychlorinated biphenyls in human serum. J Chromatogr B Analyt Technol Biomed Life Sci 852:15–21; doi:10.1016/j.jchromb.2006.12.049. PMID: 17267299.

Haug LS, Sakhi AK, Cequier E, Casas M, Maitre L, Basagana X, et al. 2018. In-utero and childhood chemical exposome in six European mother-child cohorts. Environ Int 121:751–763; doi:10.1016/j.envint.2018.09.056. PMID: 30326459.

Haug LS, Thomsen C, Becher G. 2009. A sensitive method for determination of a broad range of perfluorinated compounds in serum suitable for large-scale human biomonitoring. J Chromatogr A 1216:385–393; doi:10.1016/j.chroma.2008.10.113. PMID: 19026423.

Koponen J, Rantakokko P, Airaksinen R, Kiviranta H. 2013. Determination of selected perfluorinated alkyl acids and persistent organic pollutants from a small volume human serum sample relevant for epidemiological studies. J Chromatogr A 1309:48–55; doi:10.1016/j.chroma.2013.07.064. PMID: 23972455.

Maitre L, de Bont J, Casas M, Robinson O, Aasvang GM, Agier L, et al. 2018. Human Early Life Exposome (HELIX) study: a European population-based exposome cohort. BMJ Open 8:e021311; doi:10.1136/bmjopen-2017-021311. PMID: 30206078.

Manzano-Salgado CB, Casas M, Lopez-Espinosa M-J, Ballester F, Basterrechea M, Grimalt JO, et al. 2015. Transfer of perfluoroalkyl substances from mother to fetus in a Spanish birth cohort. Environ Res 142:471–478; doi:10.1016/j.envres.2015.07.020. PMID: 26257032.

Philippat C, Mortamais M, Chevrier C, Petit C, Calafat AM, Ye X, et al. 2012. Exposure to phthalates and phenols during pregnancy and offspring size at birth. Environ Health Perspect 120:464–470; doi:10.1289/ehp.1103634. PMID: 21900077.

Poothong S, Lundanes E, Thomsen C, Haug LS. 2017. High throughput online solid phase extraction-ultra high performance liquid chromatography-tandem mass spectrometry method for polyfluoroalkyl phosphate esters, perfluoroalkyl phosphonates, and other perfluoroalkyl substances in human serum, plasma, and whole blood. Anal Chim Acta 957:10–19; doi:10.1016/j.aca.2016.12.043. PMID: 28107829.

Ramon R, Murcia M, Aguinagalde X, Amurrio A, Llop S, Ibarluzea J, et al. 2011. Prenatal mercury exposure in a multicenter cohort study in Spain. Environ Int 37:597–604; doi:10.1016/j.envint.2010.12.004. PMID: 21239061.

Rodushkin I, Ödman F, Olofsson R, Axelsson MD. 2000. Determination of 60 elements in whole blood by sector field inductively coupled plasma mass spectrometry. J Anal At Spectrom 15:937–944; doi:10.1039/B003561K.

Sabaredzovic A, Sakhi AK, Brantsæter AL, Thomsen C. 2015. Determination of 12 urinary phthalate metabolites in Norwegian pregnant women by core-shell high performance liquid chromatography with on-line solid-phase extraction, column switching and tandem mass spectrometry. J Chromatogr B Analyt Technol Biomed Life Sci 1002:343–352; doi:10.1016/j.jchromb.2015.08.040. PMID: 26355271.

Sakhi AK, Sabaredzovic A, Papadopoulou E, Cequier E, Thomsen C. 2018. Levels, variability and determinants of environmental phenols in pairs of Norwegian mothers and children. Environ Int 114:242–251; doi:10.1016/j.envint.2018.02.037. PMID: 29524920.

Valvi D, Monfort N, Ventura R, Casas M, Casas L, Sunyer J, et al. 2015. Variability and predictors of urinary phthalate metabolites in Spanish pregnant women. Int J Hyg Environ Health 218:220–231; doi:10.1016/j.ijheh.2014.11.003. PMID: 25558797.
